# New Antimicrobial Potential and Structural Properties of PAFB: A Cationic, Cysteine-Rich Protein from *Penicillium chrysogenum* Q176

**DOI:** 10.1038/s41598-018-20002-2

**Published:** 2018-01-29

**Authors:** Anna Huber, Dorottya Hajdu, Doris Bratschun-Khan, Zoltán Gáspári, Mihayl Varbanov, Stéphanie Philippot, Ádám Fizil, András Czajlik, Zoltán Kele, Christoph Sonderegger, László Galgóczy, Andrea Bodor, Florentine Marx, Gyula Batta

**Affiliations:** 1 Division of Molecular Biology, Biocenter, Medical University of Innsbruck, Innrain 80-82, 6020 Innsbruck, Austria; 2 Department of Organic Chemistry, Faculty of Science and Technology, University of Debrecen, Egyetem tér 1, 4032 Debrecen, Hungary; 3 Faculty of Information Technology and Bionics, Pázmány Péter Catholic University, Práter u. 50A, 1083 Budapest, Hungary; 4 SRSMC, UMR 7565, Université de Lorraine - CNRS, Faculté de Pharmacie, 5 rue Albert Lebrun, BP 80402, 54001 Nancy, France; 5 Department of Medical Chemistry, Faculty of Medicine, University of Szeged, Dom Sq 8, 6720 Szeged, Hungary; 6 Department of Microbiology, Faculty of Science and Informatics, University of Szeged, Közép fasor 52, 6726 Szeged, Hungary; 7 Institute of Chemistry, Laboratory of Structural Chemistry and Biology, Eötvös Loránd University, Pázmány Péter sétány 1/A, 1117 Budapest, Hungary

## Abstract

Small, cysteine-rich and cationic proteins with antimicrobial activity are produced by diverse organisms of all kingdoms and represent promising molecules for drug development. The ancestor of all industrial penicillin producing strains, the ascomycete *Penicillium chryosgenum* Q176, secretes the extensively studied antifungal protein PAF. However, the genome of this strain harbours at least two more genes that code for other small, cysteine-rich and cationic proteins with potential antifungal activity. In this study, we characterized the *pafB* gene product that shows high similarity to PgAFP from *P*. *chrysogenum* R42C. Although abundant and timely regulated *pafB* gene transcripts were detected, we could not identify PAFB in the culture broth of *P*. *chrysogenum* Q176. Therefore, we applied a *P*. *chrysogenum*-based expression system to produce sufficient amounts of recombinant PAFB to address unanswered questions concerning the structure and antimicrobial function. Nuclear magnetic resonance (NMR)-based analyses revealed a compact β-folded structure, comprising five β-strands connected by four solvent exposed and flexible loops and an *“abcabc”* disulphide bond pattern. We identified PAFB as an inhibitor of growth of human pathogenic moulds and yeasts. Furthermore, we document for the first time an anti-viral activity for two members of the small, cysteine-rich and cationic protein group from ascomycetes.

## Introduction

The increasing incidence of fatal microbial infections due to the development of resistance against licensed antimicrobial drugs raises a strong demand for new antimicrobial treatment strategies. Filamentous ascomycetes are a rich source of antimicrobial bio-molecules that have the potential for wide application in medicine and agriculture to prevent and treat microbial infections^1^. As such, the industrially relevant fungus *Penicillium chrysogenum* is not only a well-known producer of the β-lactam antibiotic penicillin, but also secretes small, cysteine-rich and cationic proteins with antimicrobial activity. *P*. *chrysogenum* is an ideal producer of bio-products with beneficial potential to mankind as it is fermentable and bulk production is easy and cheap^2^. Most importantly, it is recognized as a “safe organism” by the US Food and Drug Administration.

The *P*. *chrysogenum* strain Q176 is the ancestor of all industrial strains used for penicillin production today^2,3^ and of the strains Wisconsin 54-1255^4^ and P2niaD18^5^, whose genomes were sequenced and are publicly available. *P*. *chrysogenum* Q176 secretes the antifungal protein PAF whose structural and functional properties have been extensively studied^6–8^. PAF represents a promising bio-molecule for novel antifungal drug development as it is stable against proteolytic degradation, thermo-resistant and active within a broad pH range^9^, and shows no cytotoxic effects *in vitro*^10^ or *in vivo*^11,12^. Furthermore, PAF could serve as a potential bio-pesticide in agriculture to impede infection by plant-pathogenic fungi^13^.

Genome mining in the strain Wisconsin 54-1255 for nucleotide (nt) sequences coding for other small, cysteine-rich and cationic proteins revealed that this *P*. *chrysogenum* strain harbours - apart from the *paf* gene (NCBI accession no. Pc24g00380)^14^ - two more genes that code for proteins with potential antimicrobial activity: Pc21g12970 and Pc12g08290. The product of the first gene (NCBI accession no. XP_002568323) is identical to the Pc-Arctin isolated from the arctic *P*. *chrysogenum* strain A096^15^, which represents an orthologue of the “bubble protein” isolated from the exudate of *Penicillium brevicompactum*^15,16^. The product of the second gene (GeneBank accession no. CAP80456) is orthologous to PgAFP identified in the supernatant of *P*. *chrysogenum* strain RP42C, originally isolated from dry-cured ham by Rodríguez-Martín *et al*.^17^. Thus, the genome of *P*. *chrysogenum* Wisconsin 54-1255 and its progenitor strain Q176 contain at least three genes that code for proteins with reported antifungal activity. This represents a unique chance to investigate their function within one organism.

PAF, PgAFP and Pc-Arctin have in common that they are encoded as prepro-proteins containing a signal peptide followed by a pro-sequence, both of which are removed during the protein maturation process for secretion into the culture supernatant^14,15,17^. The mature proteins are cationic due to a high content of amino acids (aa) with positively charged side-chains. Phylogenetic analyses suggested a classification of the three proteins into different groups, whereby Pc-Arctin is grouped more distantly from PAF and PgAFP^1,18^.

While the predicted PgAFP-orthologous protein from *P*. *chrysogenum* Q176 has not been isolated and characterized to date, a high thermo-, pH- and protease stability has been reported for the mature PgAFP of strain RP42. Potent growth inhibitory activity against toxigenic moulds on dry-ripened sausages renders this protein interesting for the control of unwanted food contamination^19^.

In the present study, we isolated and studied the PgAFP orthologous protein from *P*. *chrysogenum* Q176 and addressed unanswered questions concerning its 3D solution structure, antimicrobial specificity and mode of action. To distinguish between the two proteins originating from the two different *P*. *chrysogenum* strains Q176 and RP42 we named the protein from strain Q176 ***P***. *chrysogenum* **A**nti**F**ungal protein **B** (PAFB), in analogy to the nomenclature of the already extensively studied homologous protein PAF from *P*. *chrysogenum* Q176^14^. We adapted the recently described *P*. *chrysogenum*-based expression system^20^ to generate sufficient amounts of PAFB for functional and structural characterization. The use of the *paf*-deficient *P*. *chrysogenum* Q176 strain (∆*paf*) as recipient ensured optimal high yield PAFB production by avoiding co-expression of the related antifungal protein PAF. We identified a disulphide-bond stabilized and compact β-folded structure of PAFB and found - apart from a growth inhibitory activity against moulds and yeasts - anti-viral activity, which has never been reported before for any member of the small, cationic and cysteine-rich protein group from ascomycetes.

## Results

### The *pafB* gene transcription

**Figure 1:**
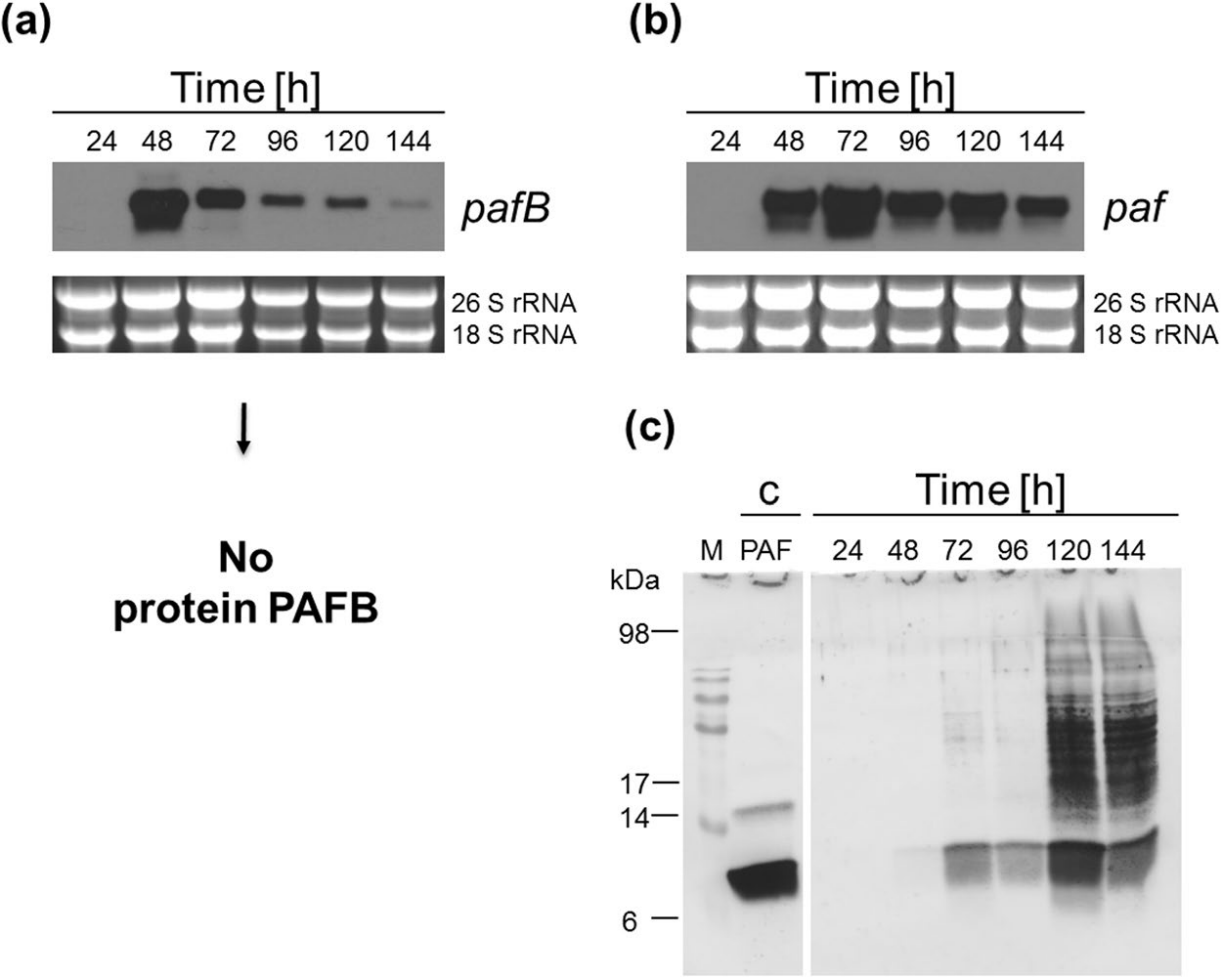
Expression of *pafB* and *paf* in *P*. *chrysogenum* Q176 submerse cultures. Samples were taken after 24, 48, 72, 96, 120 and 144 h of incubation at 25 °C. For Northern blot analysis 10 µg total RNA were loaded per lane on a 1.2% denaturing agarose gel, blotted and hybridized with a **(a)** *pafB* or **(b)** *paf* specific DIG-labelled probe. Ethidium bromide-stained 26 S and 18 S rRNA is shown as loading control. **(c)** For Western blot analysis, 25 µL of 10-fold concentrated culture supernatants were loaded per lane, size fractionated on a 18% (w/v) SDS-polyacrylamide gel and transferred on a nitrocellulose membrane. A polyclonal antibody was used for specific PAF detection. (**c**) purified PAF (1 µg) was loaded as a control. Gels and blots in (**a**), (**b**) and (**c**) were cropped for clarity. Full-length gels and blots are included in the Supplementary Information.

Our efforts failed so far to identify PAFB in the supernatant or cell extracts of *P*. *chrysogenum* Q176 grown under standard conditions (shaking culture in minimal medium MM at 25 °C for up to 6 days) (Fig. 1). The same was true for the *P*. *chrysogenum* strain ∆*paf* that lacks the PAF-encoding gene^21^. We examined this mutant to increase the chance to identify PAFB in the absence of PAF (data not shown). Therefore, we performed Northern blot analyses to determine the abundance and timing of *pafB* transcription. Surprisingly, we found that *pafB* mRNA was detectable in *P*. *chrysogenum* Q176 shaking cultures with an expression maximum at 48 h after inoculation and a subsequent decrease in transcript amount upon further incubation time (Fig. 1a).

In contrast, gene transcripts of *paf* could be detected from 48 h after inoculation onwards and transcription reached its maximum at 72 h (Fig. 1b). This expression pattern correlated with the secretion of PAF protein into the culture broth of *P*. *chrysogenum* Q176 during the stationary growth phase as reported^14^. This result was confirmed by Western blot by using a specific anti-PAF antibody^22^ (Fig. 1c, Supplementary Methods).

Incorrect mRNA processing could result in defective protein translation and the production of nonsense protein products that are readily degraded^23,24^. Therefore, we next investigated if the *pafB* mRNA was correctly spliced. The verification of the *pafB* mRNA sequence was done by cDNA synthesis, PCR amplification of the *pafB* specific transcript and subsequent Sanger sequencing. The obtained sequence matched that of the exons of the *pafB* fragment (Pc24g00380) from the *P*. *chrysogenum* Wisconsin 54-1255 genome^4^ and yielded an intact *pafB* open reading frame indicating correct splicing (Supplementary Fig. S1). Similar to the *paf* gene^14^, two short introns (63 nt and 62 nt in length, respectively) are present in *pafB* that contain the consensus 5′-splice donor site (GT) and the consensus 3′-splice acceptor site (AG). A consensus sequence for the internal putative lariat formation element (RCTRAC) can be found in the first intron, but not in the second non-coding sequence (Supplementary Fig. S1).

**Figure 2:**
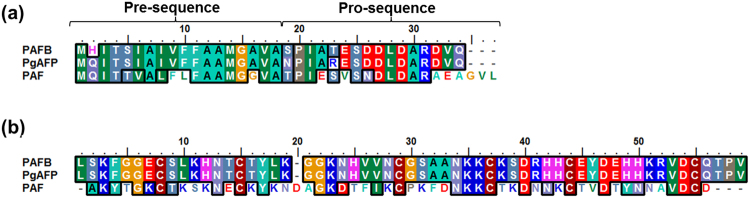
ClustalW alignment of the **(a)** prepro-sequences and **(b)** sequences of the mature proteins PAFB, PgAFP (UniProt ID: D0EXD3) and PAF (Uniprot ID: Q01701). Alignment was performed using the software BioEdit Sequence Alignment Editor.

The nt sequence of the *pafB* cDNA (deposited at European Nucleotide Archive (ENA), accession no. LT854946) codes for a 92 aa protein (Supplementary Table S1). Comparison of the PAFB primary sequence with that of the orthologous PgAFP revealed that PAFB differed in three aa, resulting in 97% identity of both proteins (Fig. 2). A histidine to glutamine substitution at position 2 resides in the pre-sequence, serine to asparagine and threonine to arginine substitutions at positions 19 and 23, respectively, occur in the pro-sequence (Fig. 2a). The predicted mature PAFB protein exhibited 100% identity with the mature PgAFP (Fig. 2b). The similarity of PAFB with PAF was 43% for the prepro-proteins and 35% for the mature proteins (Fig. 2b).

### Recombinant expression and purification of PAFB

As we could not detect and isolate PAFB from the *P*. *chrysogenum* Q176 culture supernatant, we employed the *P*. *chrysogenum*-based expression system^20^ to produce recombinant PAFB for structural analysis and functional tests. To this end, the *P*. *chrysogenum* Δ*paf* strain was transformed with a *Pst*I-linearized plasmid carrying the *pafB* gene under the control of the strong *paf* promoter^20^. Positively transformed, *ptrA*-resistant *P*. *chrysogenum* clones were tested for highest protein secretion over a time course of 96 h using small-scale fermentation (data not shown). Southern blot experiments with the best PAFB producer clone (*P*. *chrysogenum pafB*) revealed multiple random integration events of the transforming DNA into the fungal genome (Supplementary Fig. S2). This clone was selected and cultivated under large scale culture conditions.

**Figure 3:**
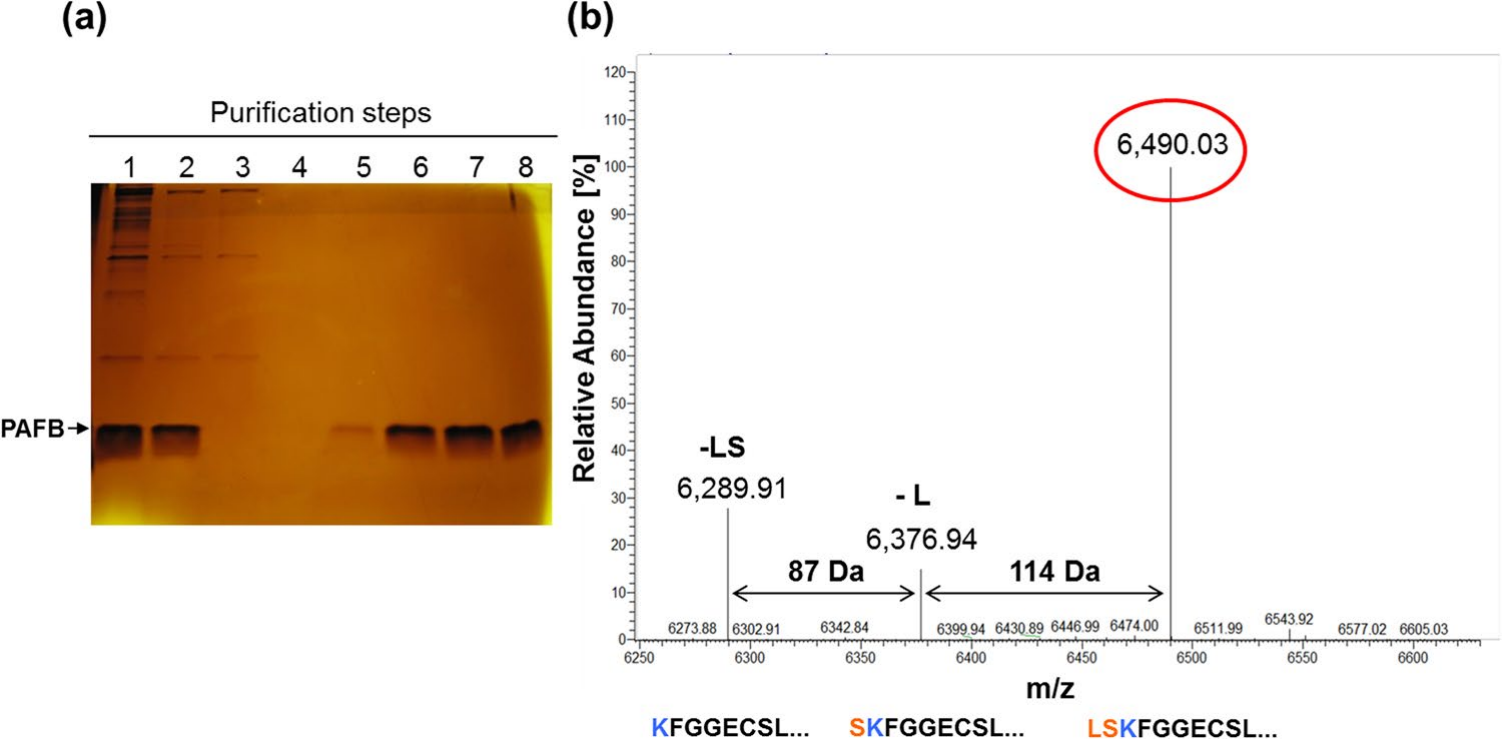
Verification of purified recombinant PAFB. **(a)** Samples of PAFB purification steps (25 µL per lane) loaded on a 18% (w/v) SDS-polyacrylamide gel and silver-stained. 1, crude supernatant of 96 h-old culture; 2, ultra-filtrated culture broth (<30 kDa); 3, flow through of cation exchange chromatography; 4, column wash fraction; 5–8, elution fractions containing pure PAFB. The gel was cropped for clarity. The full-length gel is included in the Supplementary Information. **(b)** ESI-MS spectrum of purified PAFB. The x-axis represents the ratio between mass and charge and the y-axis represents the relative abundance of isotopes. The masses correspond to the different N-terminally truncated PAFB forms: the full-length (6,490.03 Da) and the two truncated variants, lacking either one (6,376.94 Da, -Leu) or two amino acids (6,289.91 Da, -Ser/-Leu), respectively. The three aa sequences below the peaks represent the N-terminus variations of recombinant PAFB.

PAFB was purified from the cell-free culture broth by a single-step chromatography. A protein yield of 61 ± 11 mg/L supernatant was reached. Purity and identity of PAFB were verified by sodium-dodecyl-sulphate polyacrylamide gel electrophoresis (SDS-PAGE) and electrospray ionization mass spectrometry (ESI-MS), respectively (Fig. 3). On the SDS-PA gels one single band for PAFB was visible in the elution fractions (Fig. 3a). ESI-MS detected one main peak corresponding to an average mass of 6.49 kDa, which represented full-length PAFB (Fig. 3b). Two smaller peaks corresponding to 6.37 kDa and 6.29 kDa correlated with two N-terminally truncated PAFB forms: the 6.37 kDa peak corresponded to a variant without the N-terminal leucine and the 6.29 kDa peak to a form that lacked the N-terminal residues leucine and serine (Fig. 3b; Supplementary Table S1). The average molecular masses of all three peaks matched the calculated theoretical molecular masses (ProtParam tool of ExPASy)^25^ of the oxidized protein forms indicating the presence of three intra-molecular disulphide bonds in all three PAFB protein variants. They all lacked any post-translational modifications, except for the cleavage of the prepro-sequence, as reported for wild-type PAF^20^.

We used the purified recombinant PAFB to generate polyclonal anti-PAFB antibodies with the aim to identify PAFB at higher specificity and sensitivity in the supernatant and cell extract of *P*. *chrysogenum* Q176 shaking cultures by Western blot technique (Supplementary Methods). The polyclonal anti-PAFB antibodies specifically recognized recombinant PAFB, but not PAF in Western blots (Supplementary Fig. S3). However, no PAFB-specific signals could be identified in the *P*. *chrysogenum* supernatants and cell extracts at the growth conditions applied, supporting our observation that PAFB was not present at concentrations that were sufficient for detection, although its encoding gene was transcribed (Supplementary Fig. S3).

### NMR analysis

**Figure 4:**
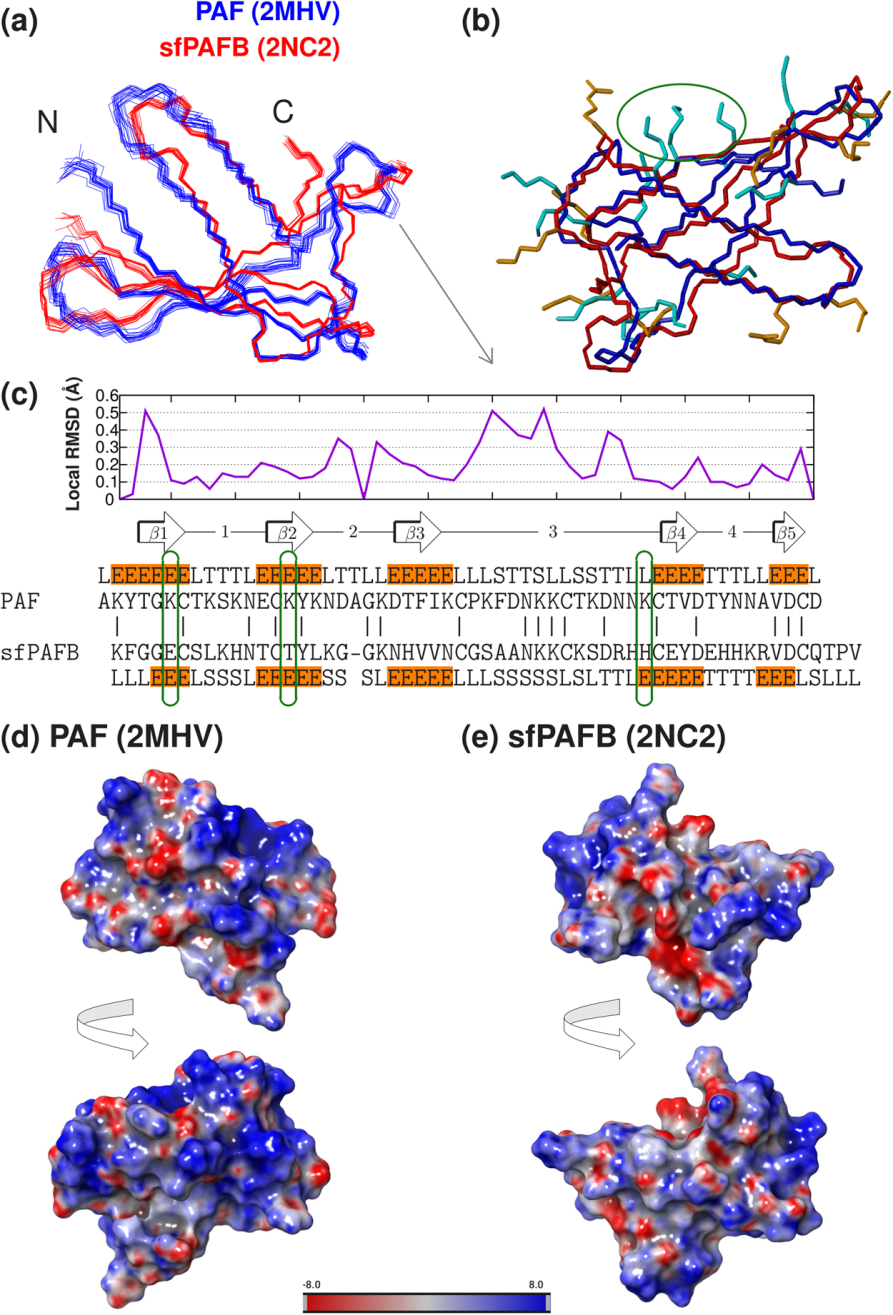
Comparison of the structures of sfPAFB and PAF. **(a)** Superimposed structure of sfPAFB (Protein Data Bank, ID: 2NC2, red) and PAF (Protein Data Bank, ID: 2MHV, blue). The N- and C-termini are indicated. **(b)** Superimposed structures with the lysine and arginine residues highlighted in stick representation (orange for sfPAFB and cyan for PAF). **(c)** Local RMSD calculated for the superimposed structures along the structure alignment generated with MAMMOTH-Mult. The position of the β-strands (β1, β2, β3, β4, β5) as assigned with DSSPcont are highlighted with arrows and orange backgrounds in the DSSPcont secondary structure labels, respectively (E for Extended, T for Turn, S for Bend, L for Loop). The three lysine residues forming a ‘belt’ in PAF are circled in green and the corresponding positions in the alignment are boxed in green. The loop regions 1–4 are indicated with lines. The arrow in light grey points out the loop region with the largest local RMSD differences. **(d)** and **(e)** Electrostatic surface potential of PAF and sfPAFB, respectively, in corresponding orientations.

To obtain structural information on PAFB with atomic resolution, solution nuclear magnetic resonance (NMR) spectroscopy experiments were performed. According to the ESI-MS data, three isoforms of unlabelled PAFB were present in solution. MS peak intensities (Fig. 3) and NMR of ^15^N-labelled PAFB suggested that the full-length PAFB sequence (58 residues) is the dominant form in the mixture. NMR spectra and MS analysis of the ^15^N/^13^C-labelled PAFB, however, identified only the shortest sequence (56 aa) (Supplementary Fig. S4). We named this short PAFB variant “short-form PAFB” (sfPAFB) and all aa sequence numbering below refers to this variant. Based on triple resonance NMR spectra, all amide H^N^ and N^H^ atoms could be found and assigned in the ^15^N-^1^H heteronuclear single quantum correlation (HSQC) spectrum of sfPAFB (Supplementary Fig. S5) except for Lys1 because the first N-terminal residue of proteins cannot be detected in the ^15^N-HSQC spectrum. Gly18, which resides in the middle of the big flexible loop 3, adjacent to Gly19 (Fig. 4), did not show a visible amide NH signal. However, other signals belonging to Gly18 were unambiguously assigned (CA and HA2, HA3). We note that for the ^15^N-labelled sample, the missing Lys1 NH signals could be found. The two overlaid ^15^N-HSQC spectra of the short (^15^N/^13^C-labelled) and full-length (^15^N-labelled) forms of PAFB exhibited good agreement for most of the peaks (Supplementary Fig. S5). As our structure calculations were based on measurements of the ^15^N/^13^C form, we decided to solve the structure of sfPAFB. As discussed below in more detail, there is no indication that the structures of PAFB and sfPAFB would exhibit differences except for the N-terminus.

The extent of backbone assignment (considering H^N^, H^N^, C’, C_α_ and H_α_ atoms in sfPAFB) reached 94.3%, and the extent of assigned protons including side chain hydrogens was 78.1%. During the ATNOS-CANDID-Cyana run 976 nuclear Overhauser effects (NOEs) were considered in the seventh cycle which resulted in an average of 17.4 NOEs/residue. The abundant distance information compared to the size of the protein resulted in a well-defined structure with very low backbone RMSD value of the final model (0.3 Å). Chemical shift data and NMR experimental parameters were submitted to the BMRB (entry no. 26001) and the structure model of sfPAFB was deposited in the Protein Data Bank (ID: 2NC2).

The structure of sfPAFB contains two orthogonally packed triple-stranded β-pleated sheets (Fig. 4). It should be noted that there are only five strands present in the protein, because the first β-strand is shared by the sheets. They are located between residues Gly4 and Cys6, Thr12 and Leu16, Asn21 and Asn25, His40 and Asp44 as well as Arg49 and Asp51, respectively. The strands are connected by three small loops (1, 2, 4) and one large loop (3) comprising β-turn motives (Fig. 4). Finally, the two β-pleated sheets have the following β-strand pattern: β1-β2-β3 and β1-β4-β5, respectively. There are six highly conserved cysteines in the amino acid sequence of sfPAFB in the form of three disulphide bonds between the cysteines 6 and 34, 13 and 41 as well as 26 and 52. Note that this pattern (*abcabc*) creates a hidden central core region. While loops 1 and 4 form turn conformations, the secondary structure analysis showed the presence of a bend in the flexible glycine-glycine motives containing loop 2. The second and third β-strands are relatively hydrophobic compared to the other parts of the protein having no charged residues. In contrast, the central part of loop 3, the so-called large loop, contains three (32, 33 and 35) conserved lysine residues, which, together with Lys9, form a positively charged surface region. In general, the large loop adopts a bent conformation, but at its C-terminal end (Asp37-Arg38) a β-turn structure is formed. The side-chain of Asp37 is close to those of Arg38 and Lys9, respectively. Interestingly, strand β4 in the middle of the second β-sheet exhibits high negative charge density. Nevertheless, there are several basic amino acids in its vicinity that potentially also form salt-bridges. Although both strands, β1 and β5, are relatively short, several H-bonds can be found between them and strand β4, stabilizing the second β-sheet. The side-chains of Tyr43 and Tyr15 interact with each other, and together with other apolar amino acids they form a hydrophobic core in sfPAFB. Finally, after the fifth, amphipathic β-strand, the C-terminal region of sfPAFB does not adopt any particular regular secondary structure.

Despite the relatively low sequence similarity (35.2%), the structure of sfPAFB is highly similar to that of PAF determined previously (Protein Data Bank ID: 2MHV). Both proteins adopt a Greek key super-secondary structure and β-barrel global fold with identical disulphide bond pattern (*abcabc*). In order to compare their local conformation, the structures of the two proteins were superimposed. Their RMSD values were below 0.5 Å overall and 0.2 Å in the β-strands (Fig. 4) except for the N-terminus. However, it turned out that the conformation of loop 3 also shows substantial differences between PAF and sfPAFB. This was unexpected because the second half of loop 3 is one of the most highly conserved regions in these proteins with the longest patch of conserved residues (asparagine-lysine-lysine-cysteine) located here (Fig. 4), whereas the β-strands generally show a lower level of sequence conservation. While both of the proteins adopt a bent structure in this region, in PAF it resulted in a turn-like conformation both at residues Asp32-Asn33 and Asp39-Asn40 amino acids, respectively. In contrast, only the latter turn is present in sfPAFB at positions Asp37-Arg38. It should also be noted that although the high density of positive surface charges is believed to be a major contributor to the activity of PAF^8^, only four lysine residues are conserved between PAF and PAFB. Furthermore, PAFB lacks a ‘belt’ of structurally aligned lysine residues (Lys 6, 15, 42 in PAF) and has generally lower overall positive surface charge and a different surface electrostatic potential (Fig. 4). To assess the possible differences between sfPAFB and the full-length protein, we built a first-approximation structural model of full-length PAFB by adding a leucine-serine segment to the N-terminus of the experimental sfPAFB structure. Short molecular dynamics (MD) runs suggest that the full-length N-terminus might align to strand β4 (residues 41–45) to form an N-terminal β-strand longer than in the sfPAFB form similar to the case of PAF (Supplementary Fig. S6). This is also plausible based on the favourable interaction between Lys1 and Asp42 (sfPAFB numbering) formed during the MD calculations. Nevertheless, we do not expect that the structures of sfPAFB and full-length PAFB exhibit substantial differences. Finally, the variation of the N-terminus does not dramatically change the biophysical features of the PAFB forms, such as isoelectric point (pI), net charge and the grand average of hydropathy (GRAVY) (Supplementary Table S1).

### Antifungal activity of PAFB

The growth inhibitory activity of PAFB was tested on filamentous fungi, yeasts and bacteria in 96-well microtiter plate assays by determination of the minimal inhibitory concentration (MIC) (Table 1). PAF was used as a control and for comparison. Among the filamentous fungi tested, the model organism *Neurospora crassa* showed the highest PAFB-sensitivity (MIC 0.12 µM in 0.1 × PDB), followed by the opportunistic human pathogens *Aspergillus fumigatus* (MIC 0.25 µM), *Trichophyton rubrum* (MIC 0.5 µM), and *Aspergillus terreus* (1 µM). Interestingly, low doses of PAFB (0.5 µM) had self-inhibition activity on the producing strain *P*. *chrysogenum* Q176 in 0.1 × PDB. This stands in contrast to PAF, which did not affect the germination of *P*. *chrysogenum* at the concentrations tested.

**Table 1:** Minimal inhibitory concentrations (MIC) of PAFB and PAF on fungi and bacteria.

Organisms	MIC [µM]	
	PAFB	PAF
Filamentous fungi		
*A*. *fumigatus*	0.25	1
*A*. *niger*	0.50	0.25
*A*. *terreus*	1	32
*N*. *crassa*	0.12/0.25^§^	0.06/0.06^§^
*P*. *chrysogenum*	0.50	>32
*T*. *rubrum*^*#*^	0.5	0.25
Yeasts		
*C*. *albicans*	1	4
*S*. *cerevisiae*	1	2
Bacteria		
*E*. *coli*	>32	>32
*B*. *subtilis*	>32	>32

^§^0.2 × Vogel’s medium.

^#^Due to slow proliferation rate the MIC of *T*. *rubrum* was determined after 8 days of incubation.

So far, no anti-yeast activity has been reported for PgAFP in malt extract broth^19^ and for PAF in YPD medium^26^. We found, however, a clear inhibitory activity for PAFB and PAF at MICs of 1 µM and 2–4 µM on the model yeast *Saccaromyces cerevisiae* and the opportunistic human pathogen *Candida albicans*, respectively, when cultivated in 0.1 × PDB. In contrast, no anti-bacterial activity of PAFB could be observed in the administered concentration range (0–32 µM) on the Gram-negative *Escherichia coli* and the Gram-positive *Bacillus subtilis*, which is similar to what is found for PAF.

PAFB efficiently prevented the conidial germination of all tested filamentous fungi at the respective MIC, except for *N*. *crassa*. Similar to previous observations with PAF^27^, *N*. *crassa* conidia still germinated in the presence of PAFB, but the elongation of the germ tubes was inhibited irrespective of the culture medium applied (0.1 × PDB or 0.2 × Vogel’s). The growth inhibitory activity of PAFB was further characterized for *N*. *crassa* 0.2 × Vogel’s medium, in which PAFB exhibited a comparable MIC as in 0.1 × PDB (Table 1). *N*. *crassa* conidia exposed to 0.25 μM PAFB for 6 h in 0.2 × Vogel’s medium germinated at a significantly lower rate (53.14 ± 5.70%, p < 0.0001) than the untreated control (83.30 ± 2.69%) (Table 2). PAF-exposure, by contrast, did not influence the germination efficiency, as 85.56 ± 1.07% of the conidia germinated in the presence of 0.06 µM PAF compared to the untreated control (Table 2).

**Table 2:** The effect of the MIC of PAFB and PAF on the colony establishment of *N*. *crassa*.

	Germination efficiency [%]^a,b^	Germ tube length [µm]^b^
control	83.30 ± 2.69	60.32 ± 10.04
PAFB	53.14 ± 5.70**	17.99 ± 2.41**
PAF	85.56 ± 1.07	48.22 ± 7.34*

^a^The germination efficiency is indicated in (%) compared to the total conidial count used, which was set to be 100%. ^b^The values are given as mean ± SD (n = 3). Significant differences (p-values) between values were determined by comparison with the untreated control.*p ≤ 0.05, **p < 0.0001.

Furthermore, PAFB significantly reduced the germ tube length of 6 h old germlings to 17.99 ± 2.41 µm at its MIC, which corresponded to a reduction of −70% compared to the untreated control (60.32 ± 10.04 µm, p < 0.0001). By contrast, PAF reduced the germ tube length less severely at its MIC (48.22 ± 7.34 µm; p ≤ 0.05; −20% compared to control) (Table 2). The increase of the PAFB and PAF concentration to 2 × MIC, respectively, did not further aggravate these effects on *N*. *crassa* (data not shown). Thus, our findings indicated that PAFB affects colony establishment of *N*. *crassa* more effectively than PAF.

It is of great relevance that an antifungal drug acts fungicidal rather than fungistatic to reduce the risk of resistance development, which occurs more readily with drugs showing fungistatic activity. We therefore tested the fungicidal potential of PAFB and PAF on the wide-spread human opportunistic pathogen *C*. *albicans*, which causes candidiasis. To this end, *C*. *albicans* cells were incubated with the antifungal proteins at concentrations corresponding to 0.25–4 × MIC for 0 h (control), 6 h and 24 h in 0.1 × PDB and were then plated in appropriate dilutions onto PDA plates to allow surviving cells to establish colonies.

**Figure 5:**
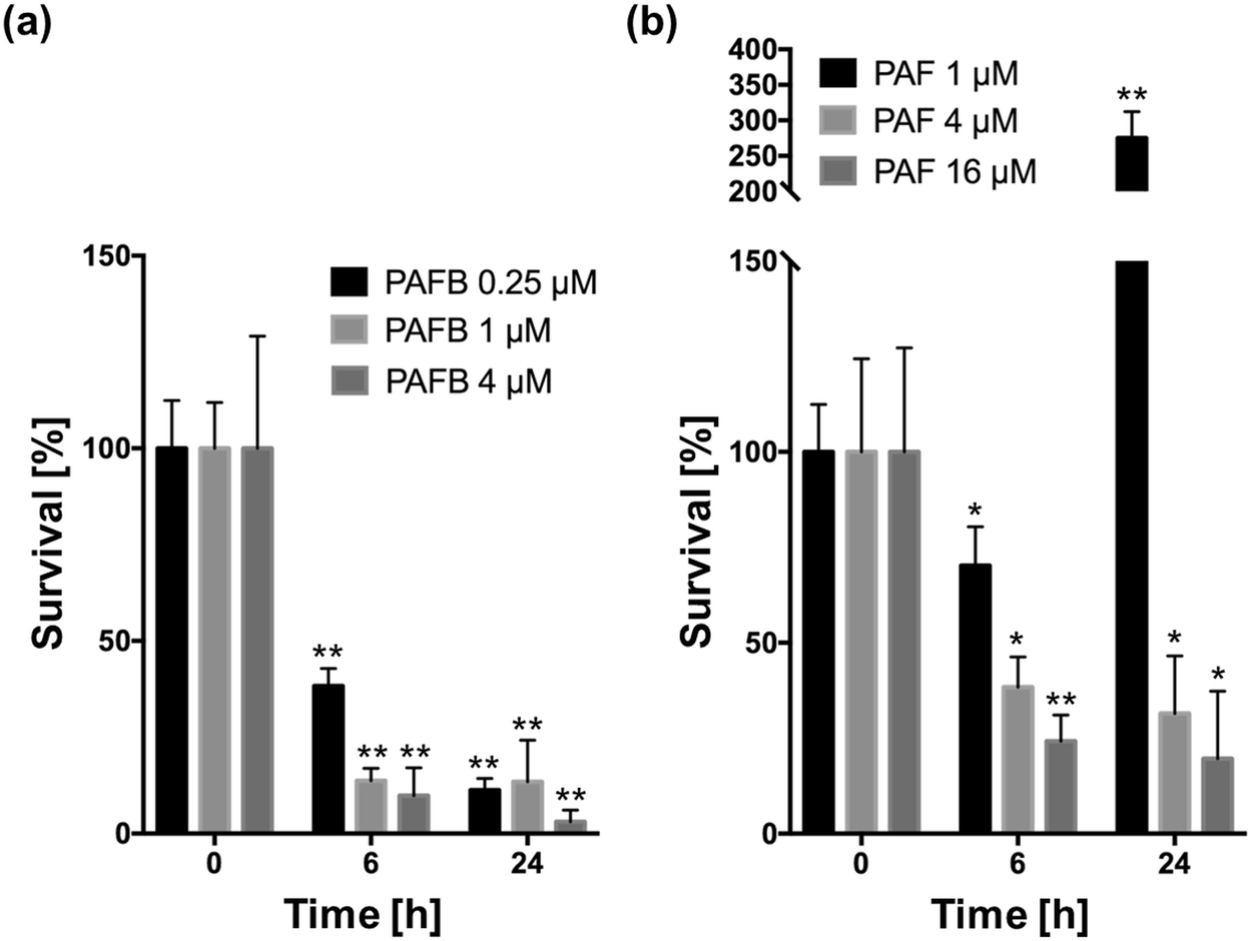
Antifungal effect of PAFB and PAF on *C*. *albicans*. Cells were exposed to 0.25 × MIC, 1 × MIC and 4 × MIC of **(a)** PAFB and **(b)** PAF. Samples at appropriate dilutions were plated on 0.1 × PDA after 0 (control), 6 and 24 h of incubation and the number of colony forming units was determined after 24 h. Colony numbers counted without incubation (0 h) were set as 100%. Values represent the mean ± SD (n = 6). Significant differences (p-values) between values were determined by comparison with the respective survival rate at 0 h. *p < 0.001, **p < 0.0001.

As shown in Fig. 5a, viability was significantly reduced after 6 h of incubation in the presence of PAFB in a dose-dependent manner. The survival rate was 38.33 ± 4.49% (p < 0.0001) at 0.25 µM, 13.74 ± 3.16% (p < 0.0001) at 1 µM and 9.81 ± 7.24% (p < 0.0001) at 4 µM compared to the viability of the respective controls (0 h of incubation) which were set to be 100%, respectively. The fungicidal potential was further increased when *C*. *albicans* cells were exposed to PAFB for 24 h. Compared to the survival rate of the controls (100%), only 11.24 ± 3.07% (p < 0.0001), 13.45 ± 10.79% (p < 0.0001) and 3.08 ± 2.99% (p < 0.0001) cells survived when exposed to 0.25, 1 and 4 µM, respectively (Fig. 5a).

Similarly, *C*. *albicans* cells exposed to PAF showed reduced viability after 6 h of incubation (Fig. 5b). The fungicidal effect of PAF, however, was less pronounced than that with PAFB. The survival rate was 70.21 ± 10.17% (p < 0.001) at 1 µM PAF, 38.35 ± 7.92% (p < 0.001) at 4 µM PAF and 24.21 ± 6.88% (p < 0.0001) at 16 µM PAF, which corresponded to 0.25 × MIC, 1 × MIC and 4 × MIC, respectively, compared to the control cells (100% survival at 0 h of incubation) (Fig. 5b). This fungicidal effect was detectable with 4 µM and 16 µM PAF at longer incubation times (survival rates after 24 h: 31.48 ± 15.05% (p < 0.001) and 19.65 ± 17.68% (p < 0.001), respectively) (Fig. 5b), whereas PAF was less effective at low concentration (0.25 × MIC) and with shorter incubation times (6 h). *Candida* cells exposed to 1 µM PAF proliferated further when cultivated for 24 h in liquid 0.1 × PDB, which became evident from the increased number of colony forming units when plated on PDA plates.

Our results indicate that PAFB acts fast and efficiently in a fungicidal way on *C*. *albicans* at a sub-MIC concentration.

### Localization of PAFB in fungi

In previous studies, we reported that PAF toxicity in sensitive fungi is closely linked with its active internalization into the cell^8,22^. Therefore, we examined the uptake and localization of PAFB in comparison to PAF in *N*. *crassa*, *P*. *chrysogenum* and *C*. *albicans*. For this purpose, PAFB and PAF were labelled with the green fluorescent dye BODIPY. Both proteins had comparable labelling efficiencies and the antifungal activity was similar to that of the unlabelled proteins (data not shown). To visualize the fluorescence signals 8 µM of protein conjugates were applied.

**Figure 6:**
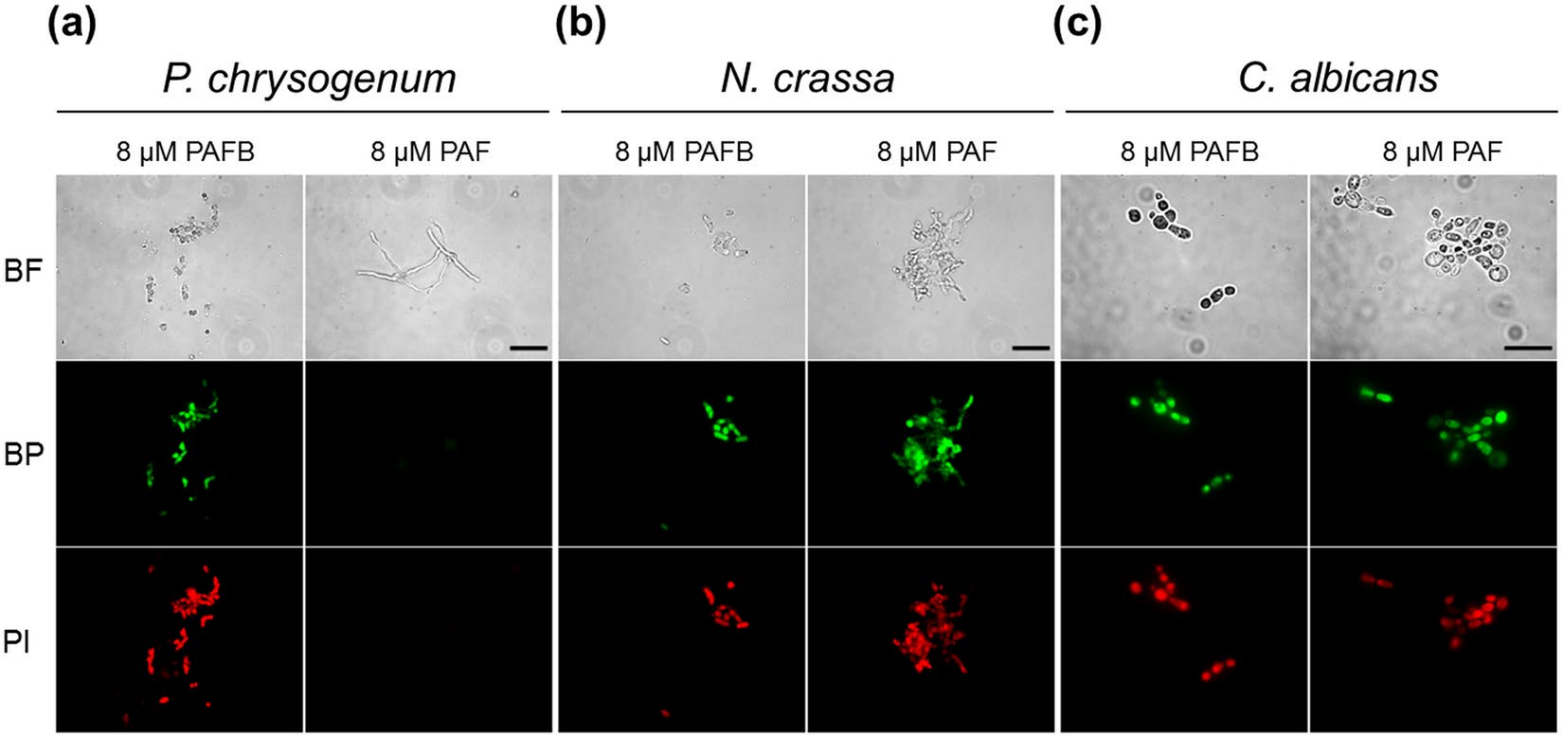
Uptake of BODIPY-PAFB and BODIPY-PAF in germlings of **(a)** *P*. *chrysogenum*, **(b)** *N*. *crassa* and **(c)** *C*. *albicans* cells. Samples were taken after 12 h of incubation of conidia of *P*. *chrysogenum* and *N*. *crassa* and after 6 h of incubation of *C*. *albicans* cells with BODIPY-labelled proteins. Co-staining with PI was applied 10 min before imaging. BF = Brightfield, BP = BODIPY-labelled proteins. Scale bars = 30 µm in (a,b), scale bar = 15 µm in (c).

The conidia of *N*. *crassa* and *P*. *chrysogenum* were allowed to germinate for 12 h, the cells of *C*. *albicans* to grow for 6 h in the presence of BODIPY-PAFB and BODIPY-PAF, respectively, before the fungal cells were co-stained with propidium-iodide (PI) to test cell viability. As shown in Fig. 6(b,c), both BODIPY-labelled proteins localized in germlings of *N*. *crassa* and in *C*. *albicans* cells, which undoubtedly showed signs of cell death by positive PI staining. The same was observed with *P*. *chrysogenum* germlings, in which BODIPY-PAFB uptake correlated with PI staining. By contrast, we did neither detect BODIPY-PAF uptake nor PI staining in *P. chrysogenum* (Fig. 6a). These observations matched with the different susceptibilities of *P*. *chrysogenum* for PAFB and PAF described above (Table 1) and provide further evidence that antifungal toxicity of PAFB and PAF is closely linked to their uptake into fungal cells.

**Figure 7:**
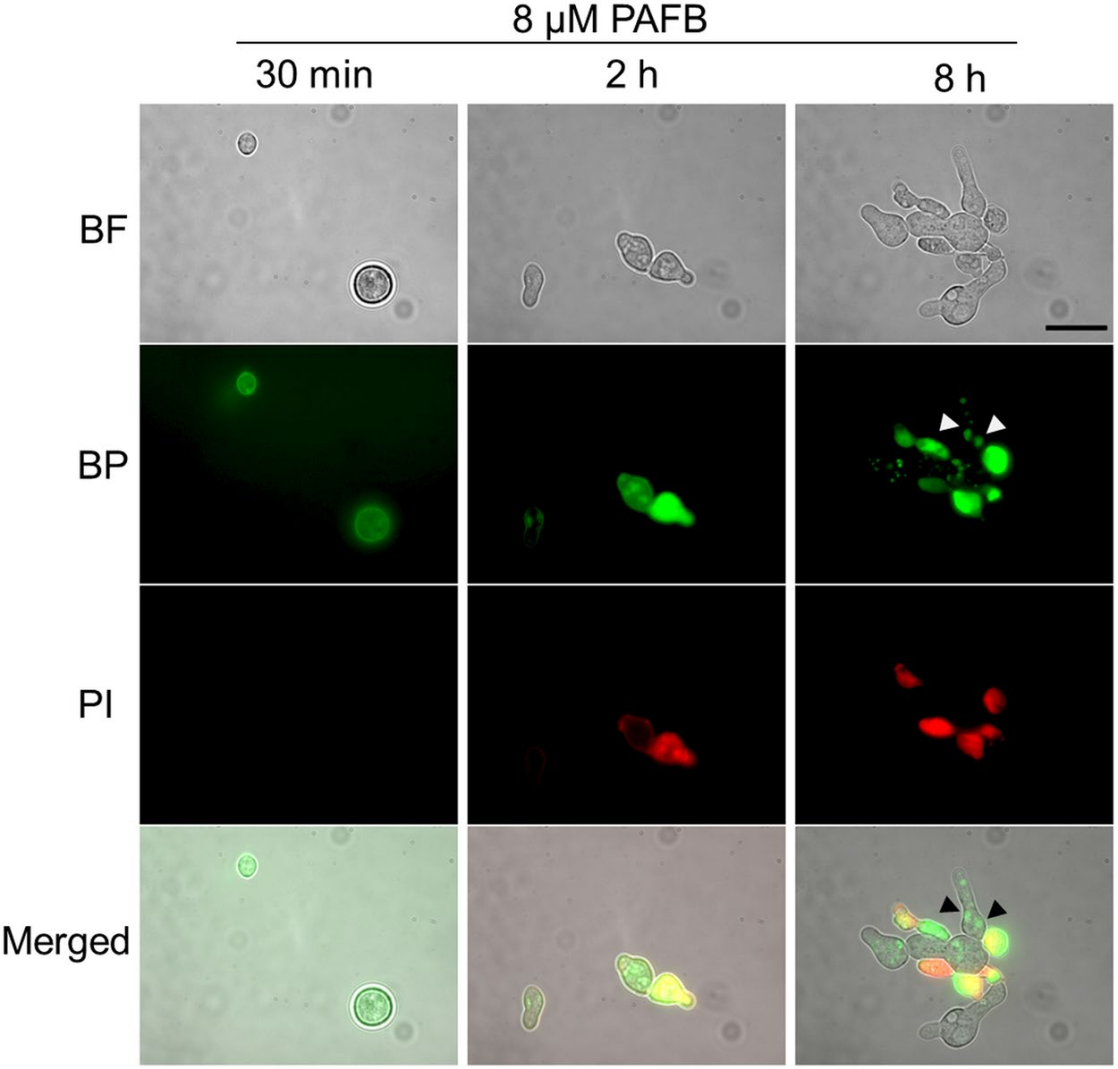
Uptake of BODIPY-PAFB (8 µM) in *N*. *crassa* conidia or germlings. Samples were taken after 30 min, 2 h and 8 h and co-stained with PI for 10 min before imaging. White and black arrows indicate BP-PAFB specific signals in vacuoles. BF = Brightfield, BP = BODIPY-labelled PAFB. Scale bar = 15 µm.

We further analysed the uptake mechanism of BODIPY-PAFB in *N*. *crassa* in time course experiments. After 30 min of incubation, BODIPY-PAFB had already attached to the envelope of *N*. *crassa* conidia and intracellular signals were visible (Fig. 7). With prolonged incubation time (>2 h), the PAFB signals in the cytoplasm steadily increased which coincided with positive PI staining (Fig. 7). Interestingly, fungal cells showed no signs of cell death as long as BODIPY-PAFB resided in vacuoles (Fig. 7).

We investigated in the next step whether PAFB enters the cells actively or passively, e.g. by diffusion. To this end, we exposed 4.5 h-old germinated conidia to NaN_3_ and carbonyl cyanide *m*-chloropheylhydrazone (CCCP), which are inhibitors of oxidative phosphorylation and ATP production, respectively, for 30 min before BODIPY-PAFB was added for further 1.5 h of incubation.

**Figure 8:**
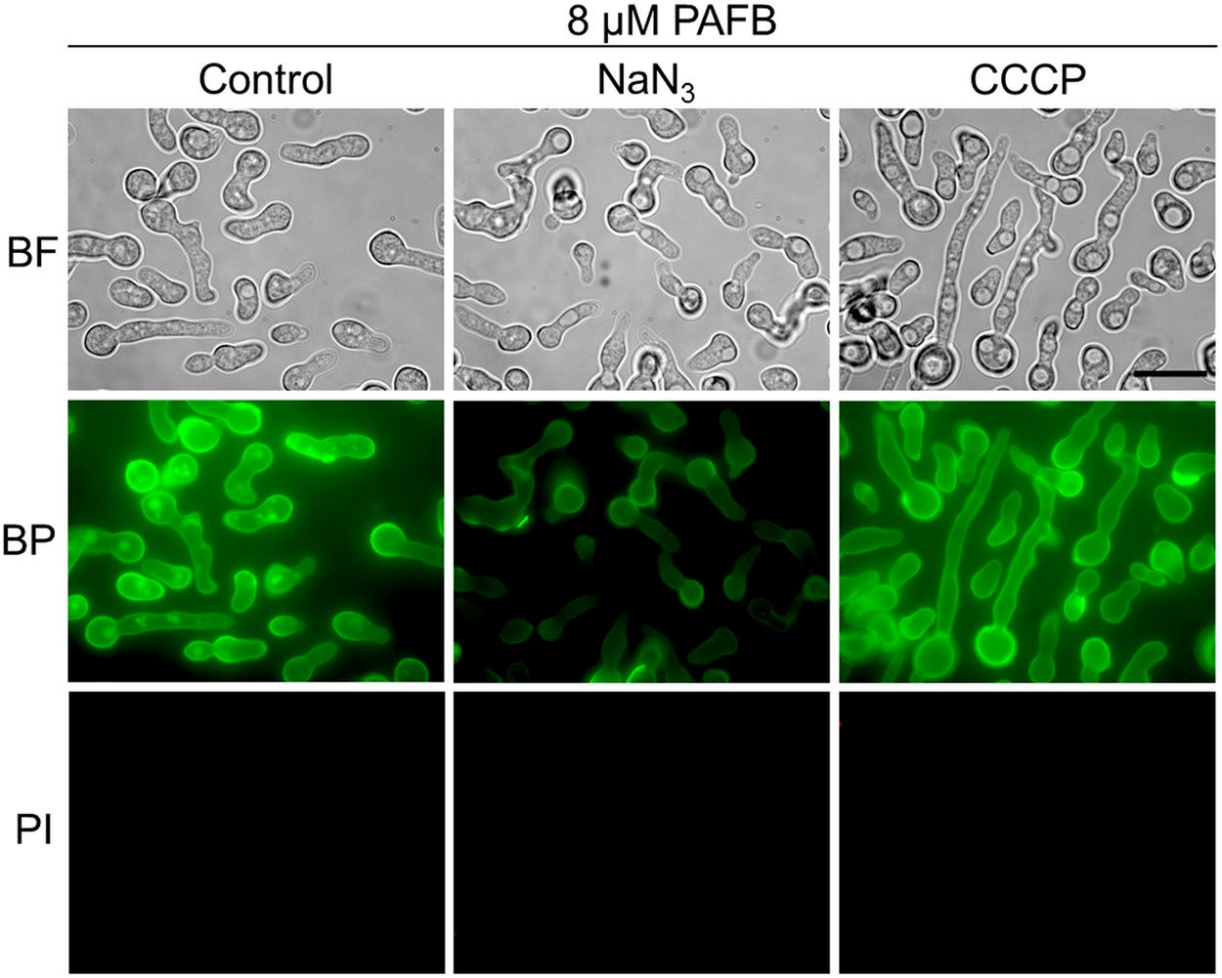
Uptake of BODIPY-PAFB (8 µM) in *N*. *crassa* germlings in the presence of 100 µM CCCP or 2.5 mM NaN_3_. The control was stained with BODIPY-PAFB without inhibitors. All samples were co-stained with PI for 10 min before imaging. BF = Brightfield, BP = BODIPY-labelled PAFB. Scale bar = 15 µm.

As shown in Fig. 8, BODIPY-PAFB remained attached to the outer layers and was not taken up into the *N*. *crassa* cells in the presence of the inhibitors (Fig. 8). In contrast, BODIPY-PAFB localized in the vacuoles of germlings that were not exposed to the inhibitors (Fig. 8). PAFB uptake was also inhibited at 4 °C, which reduces cell metabolism (data not shown).

These observations proved the staining specificity of BODIPY-PAFB and indicate that the internalization of BODIPY-PAFB depends on an active cellular metabolism. Negative PI staining confirmed the viability of the cells under the applied test conditions.

### Antiviral activity

**Figure 9:**
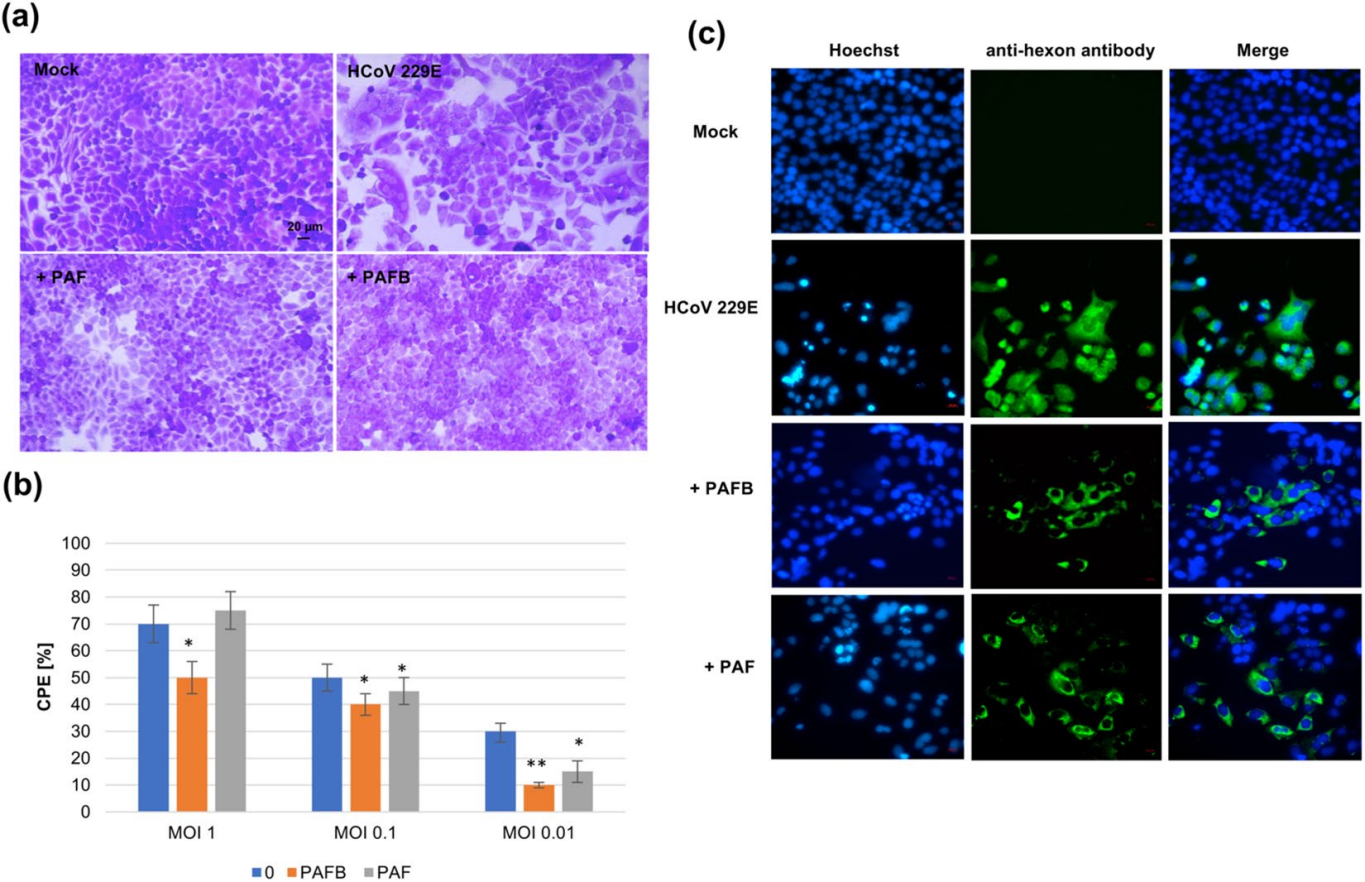
PAFB and PAF reduced CPE of HCoV 229E in L132 cells. PAFB and PAF (8 µM, respectively) were applied. **(a**) Crystal violet assay visualized the reduction of virus-induced CPE at MOI 0.01. **(b)** Reduction of the viral CPE at MOI 1, MOI 0.1 and MOI 0.01 at 72 h post infection in the presence of PAFB and PAF (0, untreated control). **(c)** Immunostaining of HCoV protein in L132 cells with mouse-anti hexon antibody and goat anti-mouse FITC-labelled secondary antibody after treatment with PAFB and PAF. Nuclei of fixed L132 cells were counter-stained with Hoechst 33342. Virus-uninfected cells (Mock) served as a negative control and virus infected cells without protein treatment as positive control (HCoV 229E); *p < 0.05; **p < 0.01.

Recently, Holthausen *et al*. (2017) reported about the efficacy of host defense peptides from the skin of the South Indian frog to treat respiratory viral infections, as in the case of human influenza A viruses^28^. In this study, we examined the selective anti-viral potential of PAFB and PAF to reduce the infection of human epithelial cell line L132 by the human coronavirus strain 229E (HCoV 229E) *in vitro*. First, we confirmed that PAFB, similarly to PAF, showed no cytotoxic effects on the host cells in the applied concentration range (0–32 μM) (Supplementary Fig. S7). The anti-viral activity of the proteins was expressed in terms of protein concentration that protects against virus-induced cytopathogenic effects (CPE) in L132 cells, such as syncytia formation, vacuolization, cell rounding, detachment and cell death (Fig. 9a). We determined 8 μM as the minimum effective protein concentration that reduced virus-induced CPE and preserved cell monolayer integrity (Fig. 9a,b). The anti-viral efficiency could be observed in virus infected L132 cells at different viral titres (multiplicity of infection (MOI) 1–0.01) (Supplementary Fig. S8), but reached a maximum inhibition value of 21.07% with PAFB against HCoV 229E at MOI 0.01 (Fig. 9b). PAF in contrast, inhibited HCoV 229E-induced CPE less effectively than PAFB, while still exhibiting antiviral activity, especially at lower viral titers: 8 μM PAF reduced the viral CPE to 11.89% at MOI 0.01 (Fig. 9a,b). The application of higher protein concentrations (16 μM and 32 μM), however, did not further increase the anti-viral effect of PAFB and PAF (Supplementary Fig. S8). The anti-viral efficacy of both proteins was also confirmed by immunofluorescence experiments, where 8 μM PAFB and PAF successfully reduced the number of HCoV 229E infected L132 cells (Fig. 9c). HCoV 229E localization was examined with a virus-specific antibody at 72 h post infection when viral replication and transcription was underway. As shown in Fig. 9c, a strong virus-specific green fluorescent signal was observed in the majority of HCoV 229E infected cells (MOI 0.01) without PAFB or PAF treatment. The signal intensity and the number of virus infected cells significantly decreased in the presence of PAFB or PAF (Fig. 9c). This observation suggests that the antiviral effect of the proteins may reduce not only the final stages of the viral replication cycle and CPE, but also the earlier steps of the infection and/or the spread of the viral particles.

## Discussion

In this study, we provide detailed insight into the antimicrobial activity and the structure of the secreted PAFB protein from the penicillin-producing mould *P*. *chrysogenum* Q176. This protein belongs to the group of small, cysteine-rich and cationic proteins from ascomycetes. Although the PAFB encoding gene was abundantly transcribed, we could not detect the translated gene product under the cultivation conditions applied. This phenomenon is not unique. Only recently, Garrigues *et al*. (2016) reported that the identification of the genome encoded antifungal protein AfpB from *Penicillium digitatum* had failed, although the gene was transcribed at high level^18^. Instead the antifungal activity of this cryptic protein was successfully analysed by mapping the antifungal motifs of AfpB with synthetic cysteine-containing peptides^29^ before recombinant, full-length AfpB was generated recently for functional analysis applying the *P*. *chrysogenum*-based expression system^20,30^. The reason remains obscure, why the timely expressed and correctly processed *pafB* mRNA was not translated into detectable protein amounts by *P*. *chrysogenum* Q176. As conidia of *P*. *chrysogenum* were PAFB susceptible, it seems that the production of PAFB needs strict regulation to avoid self-limitation. On the other hand, the pre-existing pool of mRNA may serve as a reservoir allowing for fast availability in case of urgent needs, e.g. a fast response to changing environmental or developmental stimuli. Indeed, a function of cysteine-rich, cationic proteins beyond fungal growth inhibition, such as stress response and asexual development, supporting the fitness of the producing moulds has been reported earlier^21,31^. A recently published transcriptome meta-analysis for the expression regulation of the *Aspergillus niger* antifungal protein AnAFP further underlines the role of these proteins in stress response, growth and development, nutrient recycling, and autophagy^32^. Thus, the *pafB* mRNA may be stored, for example in processing bodies or stress granules as described for *S*. *cerevisiae* and mammalian cells, until the PAFB protein is needed^33–35^. We are currently investigating this assumption.

We applied the *P*. *chrysogenum*-based expression system to generate PAFB in sufficient quantity and high quality for functional and structural analyses. The strong *paf* promoter, the presence of multiple gene copies and/or possibly the genomic integration site are responsible for the bulk production of recombinant PAFB^20,36^. The fact that recombinant PAFB was produced in *P*. *chrysogenum* ∆*paf* without problems indicates that regulatory elements, likely present in the 5’- and/or 3’- regions of the *pafB* mRNA and responsible for controlling mRNA translation and/or storage in the wild-type, were replaced in the *pafB* expression cassette.

Mature PAFB was purified in a major full-length form with 58 aa, and two less abundant short variants with 57 aa and 56 aa, respectively. Another member of this protein group, the *Aspergillus giganteus* AFP, is secreted in two naturally occurring N-terminal variants, the major short form (51 aa) and in lower amounts the larger form (lf-AFP), containing six additional N-terminal amino acids^37^. Lf-AFP remarkably differs from AFP in pI, GRAVY and net charge and shows lower antifungal activity^37,38^. To date, we cannot tell whether the N-terminal PAFB variants occur naturally, e.g. by autocatalytic activities, or whether they are a consequence of overexpression conditions. The latter has been observed for example, for the expression of the recombinant bovine chymosin in *Aspergillus awamori*, which resulted in products with variable N-termini, most likely due to proteolysis in the culture supernatant^39^. Recombinant PAF produced in high yields in *P*. *chrysogenum* showed no N-terminal variations whereas subtle N-terminal differences were detected when expressed in *P*. *digitatum*^20^. Similarly, AfpB expression in *P*. *digitatum* using the *paf*-derived expression cassette resulted in gene products with N-terminal variations^30^. In none of these cases, however, the antifungal activity of the N-terminal variants was affected^20,30^. Proteolysis of the PAFB N-terminus might be influenced by the nutrient composition of the fermentation broth. For double isotope labelling (^15^N/^13^C) of PAFB, sucrose was replaced with ^13^C-glucose as carbon source in the growth medium, which might be responsible for the generation of sfPAFB (56 aa). This assumption requires further analysis in the future. In one of our recent studies, we demonstrated that the N-terminal aa regulate the stability, folding and antifungal function of the recombinant NFAP protein from *Neosartorya fischeri*^40^. By contrast, we found here, that the N-terminal PAFB variants do not differ significantly in their overall solution structure and physico-chemical features. We therefore assume that the antifungal activity is not affected.

Importantly, the mature full-length PAFB is identical to the mature PgAFP described from *P*. *chrysogenum* RP42C, originally isolated from cured ham^41^. PgAFP was shown to be sensitive to the ionic strength of the fungal culture medium^42^, and highly stable under extreme pH, high temperature and against proteolysis^19^. This is also true for other members of this antifungal protein group, such as PAF, AFP, or NFAP, which possess a highly stable disulphide-bond mediated, compact, β-fold structure^9,40,43^.

PAFB showed potent antifungal activity against model fungi and human fungal pathogens in low concentrations (µM range). PAFB prevented more effectively than PAF the germination and colony establishment of *N*. *crassa*, and significant differences in the susceptibility of some tested fungi, e.g. *A*. *terreus*, *P*. *chrysogenum*, point towards different modes of action of these two proteins. Variations in the antifungal spectrum and the mode of action were reported for the structurally very similar, related antifungal proteins PAF, AfpB, NFAP and AFP^26,30,44,45^. We propose that the presence and activity of host interaction molecules that regulate the uptake mechanism, the processing of signals and the ability to cope with detrimental effects triggered by the antifungal proteins determine the susceptibility of fungal species. Furthermore, the importance of protein surface charges and their distributions described for PAF function need to be considered to explain differences in the antifungal activity^8^.

Recombinant PAFB was identified after 72 h of cultivation and reached its maximum amount after 96 h in the standard medium MM, which perfectly coincided with the onset of production of PAF and other recombinant PAF variants that had been generated with this expression system under the control of the *paf* promoter^20^. Interestingly, the overproduction of recombinant PAFB did not affect the proliferation of the producer strain *P*. *chrysogenum pafB*, although an inhibitory activity was observed on conidia in the microdilution assays (Table 1). One possible explanation for this phenomenon could be that under PAFB expression conditions (established hyphal growth and MM), *P*. *chrysogenum* hyphae lack any PAFB susceptibility determinants, which, in contrast, must be present in 0.1 × PDB. Indeed, we proved that PAFB, similarly to PAF, did not interact with *P*. *chrysogenum* hyphae cultivated in MM (Supplementary Fig. S9). Only when grown in 0.1 × PDB, hyphal proliferation was inhibited and PAFB was taken up by the cells (Supplementary Fig. S9). PAF, on the other hand, neither interacted with *P*. *chrysogenum* conidia nor hyphae, irrespective of the culture medium applied (Table 1 and Supplementary Fig. S9).

Similar nutrient dependent susceptibility for PAFB and PAF was observed with yeasts. Up to date, potent anti-yeast activity was only reported for the *N*. *fischeri* protein NFAP2, which killed yeast cells within minutes by plasma membrane disruption^1^. Neither for PgAFP^19^, nor for the related proteins AFP, NFAP and PAF any yeast inhibitory activity was ever found^1,46^. Most interestingly, in this study we show for the first time, anti-yeast activity of PAFB and PAF in 10 × diluted PDB. Apparently, yeast cells exhibit susceptibility determinants for both proteins when cultivated in this “starvation” medium. The time course experiments indicated that *C*. *albicans* cells were not killed immediately after administration of PAFB and PAF, but that cell death occurred in a time and concentration dependent manner, supporting our observations made so far that exclude a fast killing mechanism, e.g. by pore formation, but point towards more complex interaction mechanisms with the target cell^8,27^.

Indeed, cell toxicity of PAFB in all susceptible fungi studied correlated with active protein internalization and its subsequent distribution in the cytoplasm, which coincided with PI positive staining as observed with PAF and other antifungal peptides and proteins in previous studies^8,22,47–49^. Higher fungicidal activity of PAFB over that of PAF might result from differences in efficient uptake and/or reaching an effective killing concentration within the cell. Further investigations are needed to elucidate the mechanisms that regulate fungal susceptibility and killing kinetics of members of this protein group.

Few studies were published on the anti-viral activity of antimicrobial peptides and proteins expressed by animals and plants, e.g. defensins^50–52^. A surprising and novel observation for small, cysteine-rich, cationic and antimicrobial proteins of fungal origin was the efficacy of PAFB and PAF against HCoV 229E virus. This virus is highly contagious and can cause mild, cold-like respiratory illness. However, highly pathogenic HCoVs such as severe acute respiratory syndrome CoV (SARS-CoV) and Middle East respiratory syndrome CoV (MERS-CoV) predominantly infect lower airways and cause fatal pneumonia with high morbidity and mortality during an epidemic^53^. Anti-HCoV drugs and vaccines are in different stages of development, but they are still lacking today^54^. PAFB and PAF, preferentially at low MOI, reduced the virus-induced CPE of infected human L132 cells, thus promising a potential alternative therapeutic approach against human coronaviruses. Based on the data collected in this study, we suggest that PAFB and PAF directly - or indirectly by influencing the host cell metabolism - inhibit the release and spread of progeny virus particles, or destroy the HCoV virions. The observed slight differences in the anti-viral activity between PAFB and PAF may result from different virus or host cell susceptibility towards both proteins, or from differences in the protein surface charge distributions as outlined above. The observation that anti-viral efficacy could not be further increased with higher protein concentrations than 8 µM could be attributed to low bioavailability of the proteins at higher concentrations, a partial clustering or hampered crossing of bio-membranes as shown for antimicrobial proteins in previous studies^55,56^. Work is currently in progress to get detailed insight into the mode of the anti-viral action of PAFB and PAF.

Importantly, lethal activity of the potential drugs towards human cells has to be excluded and harmful side effects need to be kept to a minimum for future antimicrobial drug development. Similar to AFP and PAF^10,57^, no toxic activity on mammalian cells was detected for PAFB *in vitro* (Supplementary Fig. S7). This feature and the antifungal/antiviral potential render PAFB a promising candidate for the development of novel antimicrobial treatment strategies.

## Methods

### Strains, media and cultivation conditions

Microbial strains used in this study and growth media are listed in the Supplementary Table S2 and Table S3, respectively.

The propagation of vectors and plasmids was carried out in *E*. *coli* DH5α, grown in lysogeny broth medium (LB), supplemented with 100 µg/mL ampicillin. Yeasts were grown on potato dextrose agar (PDA, Becton Dickinson) or on solid yeast extract peptone dextrose medium (YPD) at 37 °C.

For the generation of conidia, *P*. *chrysogenum* was cultivated on solid defined minimal medium (MM) at 25 °C, *N*. *crassa* on Vogel’s agar plates at 37 °C under continuous light and *A*. *fumigatus*, *A*. *niger* and *A*. *terreus* were grown on PDA (Becton Dickinson) at 37 °C. *T*. *rubrum* was grown on oatmeal agar. Conidia were harvested in spore buffer (0.9% NaCl (w/v), 0.01% Tween 80 (v/v)). For transformation, 10^8^ conidia of *P*. *chrysogenum* Δ*paf*^21^ were grown in 200 mL CM for 38 h at 25 °C under continuous shaking (210 rpm). Conidia (1–2 × 10^8^) of *P*. *chrysogenum* Q176 and *P*. *chrysogenum pafB* were cultivated in 200 mL MM for 24–144 h at 25 °C under continuous shaking for Northern, Southern and Western blot analyses and PAFB production, respectively. PAFB single (^15^N) or double (^15^N/^13^C) isotope-labelling for NMR analysis was carried out by replacing the nitrogen and/or carbon source in MM by 0.3% Na^15^NO_3_ and/or 1% ^13^C-glucose (w/v) (Euriso-Top), respectively.

### Northern analysis

Total RNA was isolated from *P*. *chrysogenum* Q176 liquid cultures using TRI Reagent (Sigma-Aldrich). Ten µg RNA per lane were loaded on 1.2% formaldehyde-agarose gels, blotted onto Hybond-N membranes (Amersham Biosiences) and hybridized with digoxigenin (DIG)-labelled probes (Roche). The primer pair o*pafB*_fw/o*pafB*_rev and o*paf*1_fw/o*paf*_rev was used for PCR-based amplification of the *pafB* and *paf* specific hybridization probes, respectively (Supplementary Table S4).

### Fungal transformation and verification of plasmid integration

For the generation of a PAFB-overproducing *P*. *chrysogenum* strain, the *paf* deletion mutant *P*. *chrysogenum* Δ*paf* ^21^ was transformed with pSK275*pafB*. Protoplast formation and transformation of 10 µg *Pst*I linearized pSK275*pafB* was carried out according to Cantoral *et al*.^58^. Transformants were single spored three times on MM agar supplemented with 0.3–0.6 µg/mL pyrithiamine hydrobromide (Sigma-Aldrich).

Positive transformants were analysed by Southern blotting for the genomic integration of pSK275*pafB*^20^. A detailed description is given in the Supplementary Methods.

### Protein expression, purification and verification

PAFB and PAF were produced and purified from the cell-free supernatant as recently described^20^. To verify the identity of purified PAFB, the protein mass was determined by ESI-MS at the Protein Micro-Analysis Facility (Medical University of Innsbruck)^20^. In addition, protein solution was size-fractionated on a 18% (w/v) SDS-PAGE as described^20^.

### NMR analyses

Three PAFB samples were prepared for NMR studies: an unlabelled, a ^15^N-labelled and a ^15^N/^13^C-labelled as described recently^20^. Lyophilized protein was dissolved in 20 mM acetate buffer (prepared with deuterated acetic acid, pH = 4.5) and filled into Shigemi NMR sample tube (250 µl). Protein concentration was set to 1.5 mM. Protein resonance assignment was accomplished with the same strategy that was applied previously for double-labelled PAF^6^. Briefly, 2D ^15^N-HSQC served as the root for the following 3D triple resonance experiments: HNCO, HN(CA)CO, HNCA, HN(CO)CA, HNCACB, HNHA, HBHA(CO)NH and HN(CO)CACB, which were used for obtaining backbone signal assignments^59^. For resonance assignment of side chains the combination of HC(C)H COSY, HC(C)H TOCSY, HCC(CO)NH and (H)CCH-TOCSY experiments were used, and aromatic protons were identified with the aid of 2D CB(CGCD)HD and CB(CGCDCE)HE spectra. The applied experiment versions were identical with those described in our previous study^6^. To collect NOE distance restraints, ^15^N- and ^13^C-resolved 3D NOESY spectra were recorded as well as a 2D ^1^H-^1^H NOESY this latter experiment was performed on the unlabelled PAFB sample. All the above listed measurements were recorded on a Bruker Avance II 500 spectrometer equipped with TXI z-gradient probe-head. The NOESY-type experiments were repeated on a 700 MHz spectrometer with similar configuration. The higher spectral resolution of the NOESY spectra with less overlapping cross-peaks help obtaining more distant restraints for structure calculation. All spectra were acquired at 298 K and data was processed with Topspin 3.0 software. Sequential resonance assignment was performed with the aid of CARA 1.8.4 software from which chemical shift data was exported to other software. Cyana 2.1 torsion angle dynamics package^60,61^ was used for *de novo* structure determination in combination with the ATNOS-CANDID algorithm (the latter was used for peak picking and NOE assignment). Setup files for Cyana were prepared manually. Disulphide connections were defined in the Cyana input assuming *abcabc* disulphide pattern as in PAF (linking cysteine 8–36, 15–43 and 28–54). Diastereotopic protons for non-equivalent CH_2_ groups were considered as stereo-chemically ambiguous assignments. Backbone torsional angle constraints were predicted according to TALOS+^62^ and were used for the refinement of the structure model. Seven cycles of Cyana iterations were performed, and the 20 lowest energy conformers were considered as a final structure model. Chemical shift data and NMR experimental parameters were submitted to the BMRB databank and the structural ensemble was uploaded to Protein Data Bank (ID: 2NC2).

### Antibacterial and antifungal activity assays

Growth inhibition assays were performed in 96-well microtiter plates (Nunclon Delta, Thermo Scientific). Conidia or yeast cells (10^4^/mL) were incubated in 0.1 × PDB (Becton Dickinson) with increasing concentrations of PAFB (0–32 µM) in a total volume of 200 µL at 25–30 °C. Bacteria were pre-cultivated in 0.1 × LB or 0.1 × PDB to reach OD_620_ of 0.05 and then exposed to antimicrobial treatment (0–32 µM PAFB) at 37 °C. Microbial growth was monitored after 24–48 h by measuring the OD_620_ with a Fluostar Omega microplate reader (BMG Labtech) and microscopically using an inverted microscope (Leica DM IL Led, Leica Microsystems). Images were taken with an AxioCam MR digital camera (Carl Zeiss GmbH) and processed with AxioVision software (Carl Zeiss GmbH). The MIC was defined as the minimal protein concentration that inhibited growth by ≥90% compared to the untreated control which was referred as 100% growth.

The germination efficiency and germ tube length of *N*. *crassa* in the presence of PAFB at its MIC was determined as described^8^.

Fungistatic and fungicidal effects on *C*. *albicans* were determined by incubating 2 × 10^4^ cells per mL in 0.1 × PDB with 0–4 µM PAFB at 30 °C with shaking for up to 24 h. Samples were taken after 0 h, 6 h and 24 h, diluted in 0.1 × PDB and plated on PDA plates in duplicates. The number of colony forming units was determined after incubating the plates for 24–48 h at 37 °C. The survival rate was calculated by setting the colony forming units at time point 0 h as 100%.

In all experiments 0–32 µM PAF was applied for comparison. Experiments were prepared in technical triplicates and performed at least twice.

### Uptake and localization studies

The green fluorophore BODIPY FL EDA (Life Technologies) was used for protein labelling as described by Sonderegger *et al*.^8^.

To study the protein uptake into *C*. *albicans*, *P*. *chrysogenum* and *N*. *crassa*, 10^6^ yeast cells/mL or conidia/mL were incubated with 8 µM BODIPY-labelled proteins in the respective media (0.1 × PDB, 0.2 × Vogel’s) at 25–30 °C and analysed within an incubation time of 30 min to 12 h. To investigate PAFB uptake into *P*. *chrysogenum* hyphae grown in MM, 24 h old mycelia were stained with 8 µM BODIPY-labelled PAFB for 2.5 h.

Co-staining with 5 µg/mL PI for 10 min at room temperature was carried out to monitor the viability of fungal cells. For positive staining controls, samples were treated with 50% (v*/*v) ethanol for 30 min.

PAFB-transport into hyphae was investigated in *N*. *crassa*. Conidia (5 × 10^5^/mL) were grown in 0.2 × Vogel´s medium for 4.5 h at 25 °C and then further incubated at 4 °C or with 2.5 mM NaN_3_ (Sigma-Aldrich) or 100 µM CCCP (Sigma-Aldrich) for 30 min, respectively. BODIPY-labelled PAFB (8 µM) was added and the samples were microscopically analysed after 1.5 h of incubation.

Microscopic imaging was performed with a fluorescence microscope (Axioplan, Carl Zeiss GmbH), equipped with an AxioCam MR3 camera (excitation/emission filters 500/535 nm for green fluorescence, 546/590 or 565/620 nm for red fluorescence, Carl Zeiss GmbH). Image processing and editing was done with Axiovision (Carl Zeiss GmbH), GIMP (GNU Image Manipulation Program, version 2.8.20; www.gimp.org) and Microsoft Power Point (Microsoft Corp.).

## Antiviral activity assays

### Cultivation of human epithelial cell line L132

For virus propagation L132 cells were grown in antibiotic-free Dulbecco’s Modified Eagle Medium with nonessential amino acids (Thermo Fisher Scientific) complemented with 10% fetal bovine serum (FBS, Eurobio). For the antiviral assays the same medium was used, containing only 2% FBS.

### Viruses

The human coronavirus HCoV 229E was propagated and quantified in L132 cells. Initial virus titers were calculated as 50% cell culture infectious doses (CCID_50_/mL), according to Reed and Muench (1938)^63^. All virus stocks were stored at −70 °C until used.

### Antiviral tests

The antiviral activity of PAFB and PAF was determined by evaluating the reduction of the virus-induced CPE, characterized by rounding, vacuolization, syncytia formation and cell death of the cell monolayer. One day before infection, 1 × 10^4^ L132 cells/well were seeded into a 96-well culture plate. The next day, medium was replaced by fresh medium containing diluted virus suspension at MOI 0.01–1.0 was added to the cell culture, followed by the immediate addition of cell culture medium supplemented with 2% FBS and 0–32 µM PAFB or PAF. Plates were then incubated at 33 °C, 5% CO_2_, for HCoV 229E strain, and checked for virus-induced CPE after 24 h and 72 h, using an inverted light microscope AE2000 (Motic).

The antiviral activity of the proteins was determined by the crystal violet assay, adapted from Feoktistova *et al*.^64^. Antiviral activity was expressed as the percentage effective CPE-inhibitory concentration. Modification of the cell morphology and reduction of the virus-induced CPE were also microscopically determined. Three independent experiments were carried out in triplicate.

For immunofluorescence staining, L132 cells were fixed in 3.7% (v/v) formaldehyde (Sigma-Aldrich) and subsequently immersed in PBS containing 0.1% (v/v) Triton X-100 for cell permeabilization. Samples were blocked in PBS/3% BSA (w/v) (Sigma-Aldrich). Hoechst 33342 (Thermo Fischer Scientific) was used as nuclear counterstains for fixed cells. The viral capsid was detected by mouse monoclonal anti-hexon antibody (1:100) (Santa Cruz Biotechnology) diluted in PBS/3% (w/v) BSA. Primary antibodies were incubated on the fixed cells for 90 min at room temperature, washed, and the antigen-antibody complexes were detected with goat anti-mouse FITC-labelled secondary antibody (1:1,000) (Santa Cruz Biotechnology) according to Grigorov *et al*.^65^. Images were acquired using an inverted epifluorescence microscope (Axio Observer 3, Carl Zeiss GmbH). Imaging and image processing was routinely performed with ZEN software (2009, Carl Zeiss GmbH) or ImageJ (The ImageJ ecosystem: An open platform for biomedical image analysis)^66^.

### Statistical analyses

Statistical analysis was performed using Microsoft Excel 2010 software (Microsoft Corp.). Two sample t-test with equal variance and one-tailed distribution was used.

### Data availability

All data generated or analysed during this study are included in this published article (and its Supplementary Information files).

## Electronic supplementary material

Supplementary Information
