# Supplementary material for: New Antimicrobial Potential and Structural Properties of PAFB: A Cationic, Cysteine-Rich Protein from Penicillium chrysogenum Q176
Source: Sci Rep. 2018 Jan 29;8:1751. doi: 10.1038/s41598-018-20002-2 (PMC5788923; doi:10.1038/s41598-018-20002-2)
Supplement: Supplementary file 1 — Supplementary Information [file 41598_2018_20002_MOESM1_ESM.pdf]

Supplementary Information

New Antimicrobial Potential and Structural Properties of PAFB: A Cationic, Cysteine-Rich Protein from *Penicillium chrysogenum* Q176

Anna Huber[1#], Dorottya Hajdu[2#], Doris Bratschun-Khan[1], Zoltán Gáspári[3], Mihayl Varbanov[4], Stéphanie Philippot[4], Ádám Fizil[2], András Czajlik[2], Zoltán Kele[5], Christoph Sonderegger[1], László Galgóczy[1,6], Andrea Bodor[7], Florentine Marx[1*], Gyula Batta[2*]

[1]Division of Molecular Biology, Biocenter, Medical University of Innsbruck, Innrain 80-82, 6020, Innsbruck, Austria
[2]Department of Organic Chemistry, Faculty of Science and Technology, University of Debrecen, Egyetem tér 1, 4032, Debrecen, Hungary
[3]Faculty of Information Technology and Bionics, Pázmány Péter Catholic University, Práter u. 50A, 1083, Budapest, Hungary
[4]SRSMC, UMR 7565, Université de Lorraine - CNRS, Faculté de Pharmacie, 5 rue Albert Lebrun, BP 80402, 54001 Nancy, France
[5]Department of Medical Chemistry, Faculty of Medicine, University of Szeged, Dom Sq 8, 6720, Szeged, Hungary
[6]Department of Microbiology, Faculty of Science and Informatics, University of Szeged, Közép fasor 52, 6726, Szeged, Hungary
[7]Institute of Chemistry, Laboratory of Structural Chemistry and Biology, Eötvös Loránd University, Pázmány Péter sétány 1/A, 1117, Budapest, Hungary

[#]These authors contributed equally to this work.

[*]**Correspondence:**
Gyula Batta: batta@unideb.hu
Florentine Marx: florentine.marx@i-med.ac.at

Supplementary Methods

Antibody generation, SDS-PAGE and Western blotting. Polyclonal antibody production in rabbits was carried out by Eurogentec (Belgium) applying the standard (87-days) immunization protocol.
For intracellular PAFB detection, cell extracts were prepared from 24-144 h old *P. chrysogenum* Q176 mycelia. Frozen and grinded mycelia (0.5 g) were extracted with one ml protein extraction buffer (12.5 mM Tris-HCl pH 6.8; 2.0% glycerol (v/v); 0.4% SDS; 2.5% β-mercaptoethanol(v/v)). The samples were then incubated at 95 °C for 5 min and centrifuged (10,000 rpm, 10 min, 20 °C). The supernatant was collected and subjected to 18% (w/v) tris-glycine SDS-PAGE and Western blotting. For the detection of PAFB in the *P. chrysogenum* culture broth, 25 µL of 10-fold concentrated supernatant was separated by 18% (w/v) tris-glycine SDS-PAGE. Samples were transferred to nitrocellulose membranes (Bio Trace, Pall), which were blocked for 2 h in PBS/0.3% Tween (v/v)/3.0% skimmed milk powder (w/v) and then incubated overnight at 4 °C with anti-PAFB antiserum (1:1,000 in PBS/0.3% Tween (v/v)/1.0% skimmed milk powder (w/v)). After washing in PBS / 0.3% Tween (3 ×10 min) the membranes were incubated with alkaline phosphatase conjugated goat anti-rabbit secondary antibody (Sigma) diluted 1:10,000 in PBS/0.3% Tween (v/v)/1.0% skimmed milk powder (w/v) for 1 h at room temperature. The membranes were washed and protein-immunocomplexes were visualized using NBT/BCIP (Promega).

Synthesis of cDNA and verification of the *pafB* coding sequence. The GoScript Reverse Transcription System kit for cDNA synthesis from total RNA was used according to the manufacturer's instructions (Promega). The cDNA served to amplify the coding sequence (CDS) of *pafB* by PCR with the primer pair o*pafB*_fw/o*pafB*_rev (Supplementary Table S4). The PCR fragment was cloned into the plasmid pGEM-T (Promega) and the CDS was determined by Sanger sequencing at Eurofins genomics (Germany).

Construction of expression vector. The Q5 High Fidelity DNA Polymerase (New England Biolabs) was used for all PCR reactions. For PAFB overexpression the *P. chrysogenum*-based expression system was used[1]. To put the *pafB* gene under the control of the strong *paf* promoter, the *paf* gene in the expression vector pSK275*paf* was replaced by *pafB* genomic DNA that codes for the pre-pro PAFB. To this end, the *pafB*-gene (404 bp) was amplified from genomic DNA of *P. chryosgenum* Q176 using the primers *pafB*_*Bgl*II_fw/*pafB*_*Sma*I_rev (Supplementary Table S4). 1,292 bp of the *paf* 5´UTR and 386 bp of the *paf* 3´UTR were amplified using the primer pairs 5UTR_*Pst*I_fw/5UTR_*Sma*I_*Bgl*II_rev and 3UTR_*Sma*I_fw/ 3UTR_*Spe*I_rev, respectively (Supplementary Table S4). After digestion of the PCR products with the respective restriction enzymes (*Sma*I, *Bgl*II, *Pst*I, *Spe*I, New England Biolabs) the resulted fragments were ligated using the T4-DNA-Ligase (Promega). The ligation product (2,082 bp) was subsequently cloned into the *Spe*I and *Pst*I digested pSK275*paf* vector exchanging the *paf*-coding sequence and resulting in the expression vector pSK275*pafB*.

Southern blotting. For Southern blotting, genomic DNA extraction was carried out according to Zadra *et al.* (2000)[2]. *Sac*II digested DNA (2 µg per lane) was fractionated on a 0.8% (w/v) agarose gel. The DNA was transferred on Hybond-N membranes and hybridized with DIG-labelled probes, specific for the pyrithiamine (*ptrA*) resistance gene present on the plasmid pSK275*pafB*. The probes were generated from pSK275*pafB* by PCR using the oligonucleotides *ptrA*_southern_fw/*ptrA*_southern_rev (Supplementary Table S4).
The Southern blot experiments proved multiple random integration of the transforming DNA into the fungal genome (Supplementary Fig. S2). A 1.3 kb DIG-labelled PCR probe spanning a part of the *ptrA* resistance cassette was used for plasmid detection. The presence of the transforming plasmid carrying the recombinant PAFB encoding gene in the genome of *P. chrysogenum pafB* strain was proven by the detection of a 3.7 kb hybridizing fragment, which corresponded to a *Sac*II digested part of the transformed plasmid. The additional signals varied in sizes and intensities, which further proved multiple-copy random plasmid integration (Supplementary Fig. S2).

***In silico* modeling and analysis of full-length PAFB.** The full-length PAFB structure was modeled by manually building and adding a leucine-serine segment to the N-terminus of the first model in the

2NC2 structure using lsqman[3] for initial superposition and standard text editing for generating the Protein Data Bank submission of the full-length model. All subsequent calculations were performed with an in-house modified version of GROMACS 4[4]. Modifications including the handling of NOE distance restraints in a pairwise manner over multiple replicas[5] were described earlier[6] and the source files are freely available at http://users.itk.ppke.hu/~gaszo. The AMBER99SB-ILDN force field[7] was used with the GBSA implicit water model using the Onufriev-Bashford-Case method[8].
The initial model was energy minimized to the 1,000 kJ/mol/nm forrce limit using steepest descents. Short exploratory molecular dynamics calculations were run with and without NOE restraints. Restrained calculations were run using the distance restraints deposited in the Protein Data Bank for sfPAFB (Protein Data Bank ID: 2NC2) on two replicas for 100 ps with a step of 1fs and NOE force constant of 1,000 kJ/mol/nm^2. In this simulation, only the final two conformations were analyzed. Unrestrained simulations were run for 3 ns and structures were sampled every 50 ps resulting in 61 models. Secondary structure content was calculated by running DSSPcont[9] on each model and then averaging the probabilities of the structural states for each residue. The state with the highest probability was chosen, in the case of equal probabilities (50-50%), preference was given to 'E' over 'L' and 'L' over 'S'.

Comparison of PAF and sfPAFB. The structures of PAF and sfPAFB were aligned with MAMMOTH-Mult[10]. Using the corresponding residue positions form the structure alignment, the NMR ensembles of the two molecules were superimposed with MOLMOL[11] that was also used for local and global RMSD calculations as well as visualization. Electrostatic potential was generated using the APBS method[12] and visualized with Schrödinger Maestro[13].

Cytotoxicity testing. The haemolytic activity of PAFB and PAF was tested on Columbia Blood Agar plates (VWR). Ten µL of protein solution (2 µg/µL) were pipetted on sterile filter discs (6 mm) placed on the agar plates. As controls 10 µL ddH_2O (negative) or 10 µL five-fold diluted Triton X-100 (positive) were used. The plates were incubated at 37 °C for 24 h.
The cytotoxicity of PAFB and PAF was evaluated on the human epithelial cell line L132 (ATCC CCL-5) according to Mosmann (1983)[14]. The assays were performed in 96-well flat bottom tissue culture plates (Sarstedt). One hundred microliters of medium (DMEM supplemented with 2% FBS) containing proteins in increasing concentrations were added to the monolayers of L132 cells (10^4/well) in triplicates. The plates were incubated at 37 °C in a 5% CO_2 atmosphere. After 72 h, the supernatant was removed and the cells were washed with phosphate buffered saline (PBS, Invitrogen, France). Ten microliters of MTT-solution (2 mg/mL), prepared in PBS, were added to each well and the plates were incubated for 4 h at 37 °C. Then, 100 µl of SDS (100 µg/mL) was added to the wells to solubilize the MTT crystals. The plates were incubated at 37 °C for 4 h, agitated until complete crystal dissolution and evaluated by measuring the OD_{570} using a Multiskan EX 96-well plate ELISA reader (Thermo Electron Corporation). The 50% cytotoxic concentration (CC50) was then determined using trend function analysis in Microsoft Excel 2010 (Microsoft Corp.).

Activity of PAFB against *P. chrysogenum*. To determine any toxic effects of PAFB on *P. chrysogenum* mycelia, conidia (10^4/mL) were seeded in 100 µl aliquots of MM or 0.1 x PDB into 96-well plates in triplicates and incubated at 25 °C for 24 h to reach an OD_{620} of 0.1-0.2. Protein was added at increasing concentrations (0-8 µM) and the plates were further incubated for 24 h. The OD_{620} was measured spectrophotometrically to determine further proliferation. Experiments were repeated at least twice.

Supplementary Tables

Table S1. Amino acid sequence and *in silico* predicted physical and chemical properties of the mature, full-length PAFB (which is identical to PgAFP[15]) and its N-terminal short-forms PAFB-L and PAFB-LS.

Protein	Number of amino acids	Molecular mass (Da)	Theoretical pI	Net charge at pH 7.0	GRAVY
**PAFB/PgAFP**	58	6500.32	8.83	+5.2	-1.031
LSKFGGECSLKHNTCTYLKGGKNHVVNCGSAANKKCKSDRHHCEYDEHHKRVDCQTPV					
**PAFB-L**	57	6387.16	8.82	+5.2	-1.116
SKFGGECSLKHNTCTYLKGGKNHVVNCGSAANKKCKSDRHHCEYDEHHKRVDCQTPV					
**PAFB-LS**	56	6300.08	8.83	+5.2	-1.121
KFGGECSLKHNTCTYLKGGKNHVVNCGSAANKKCKSDRHHCEYDEHHKRVDCQTPV					

ExPASy ProtParam tool (Gasteiger, E. *et al.* Protein identification and analysis tools on the ExPASy server in: The proteomics protocols handbook)[16], Protein Calculator v3.4 server (The Scripps Research Institute; http://www.scripps.edu/~cdputnam/protcalc.html)

Table S2. Microbial strains used in this study.

Strain	Source
Aspergillus niger	CSB12049
Aspergillus fumigatus	ATCC 46645
Aspergillus terreus T90	Maurer *et al.* 2015[17]
Neurospora crassa	FGSC #4200
Penicillium chrysogenum Q176	ATCC 10002
Penicillium chrysogenum Δ*paf*	Hegedüs *et al.* 2011[18]
Penicillium chrysogenum pafB	this study
Trichophyton rubrum	ATCC 28188
Candida albicans	CBS 5982
Saccharomyces cerevisiae	BY4741
Escherichia coli	DH5α
Bacillus subtilis	ATCC 6633
Human coronavirus HCoV 229E	ATCC VR 740

Table S3. Media used in this study

Culture medium	Composition
**Lysogeny broth medium (LB)**	1.0% NaCl, 1.0% bacteriological peptone, 0.5% yeast extract (w/v)
**Minimal medium (MM)**	0.3% $NaNO_3$, 0.05% $MgSO_4 \times 7H_2O$, 0.05% KCl, 0.005% $FeSO_4 \times 7H_2O$, 2% D(+)-sucrose (w/v), 2.5% KPO_4-buffer (pH = 5.8), 0.1% trace elements A (v/v)
**Trace elements A**	0.1% $FeSO_4 \times 7H_2O$, 0.9% $ZnSO_4 \times 7H_2O$, 0.04% $CuSO_4 \times 5H_2O$, 0.01% $MnSO_4 \times H_2O$, 0.01% H_3BO_3, 0.01% $Na_2MoO_4 \times 2H_2O$ (w/v)
**Complete Medium (CM)**	2.0% salt solution A, 0.1% trace elements B (v/v), 2.0% D(+)-glucose (w/v), 0.2% bacteriological peptone, 0.1% yeast extract, 0.1% NZ-Amine (w/v), pH = 6.5
**Salt solution A**	2.6 % KCl, 2.6% $MgSO_4 \times 7H_2O$, 7.6% KH_2PO_4 (w/v), 0.2% chloroform (v/v)
**Trace elements B**	1.3% $ZnSO_4 \times 7H_2O$, 0.07% $CuSO_4 \times 5H_2O$, 0.1% $MnSO_4 \times H_2O$, 0.006% $Na_2B_4O_7 \times 10H_2O$, 0.13% $Na_2MoO_4 \times 2H_2O$, 0.23% $FeSO_4 \times 7H_2O$ (w/v)
**Yeast Extract Peptone Dextrose medium (YPD)**	1.0% yeast extract, 2.0% bacteriological peptone, 2.0% D(+)-glucose (w/v)
**Vogel´s medium**	2.0% salt solution B (v/v), 2.0% D(+)-sucrose (w/v)
**Salt solution B**	15.0% sodium citrate, 25.0% KH_2PO_4, 10.0% NH_4NO_3, 1.0% $MgSO_4 \times 7H_2O$, 0.1% $CaCl_2$ (w/v), 0.10% trace elements C, 0.05% biotin (v/v)
**Trace elements C**	5.0% citric acid × H_2O, 5.0% $ZnSO_4 \times 7H_2O$, 0.97% $FeSO_4 \times 7H_2O$, 0.25% $CuSO_4 \times 5H_2O$, 0.05% $MnSO_4 \times H_2O$, 0.05% H_3BO_3, 0.05% $Na_2MoO_4 \times 2H_2O$ (w/v)
**Oatmeal medium**	1.0% oatmeal powder, 0.15% KH_2PO_4, 0.1% $NaNO_3$, 0.1% 0.05% $MgSO_4 \times 7H_2O$ (w/v)

Table S4. Oligonucleotides used in this study.

Primer	Sequence 5'-3'
o*pafB*_fw	ATGCATATTACTAGCATTGC
o*pafB*_rev	TTGGTTCAAACTGGGGTC
o*paf1*_fw	GGTACCATCGCCCAAATCACCACAGTTG
o*paf*_rev	GATCGGATCCCTAGTCACAATCGACAGC
*pafB*_*Bgl*II_fw	AGATCTATGCATATTACTAGCATTGCCATTGTC
*pafB*_*Sma*I_rev	CCCGGGTCAAACTGGGGTCTGGCAG
5UTR_*Pst*I_fw	CTGCAGGAATTCAGAGAGCTTTTC
5UTR_*Sma*I_*Bgl*II_rev	CCCGGGAGATCTTATGAAGGGCTTGAGATGATG
3UTR_*Sma*I_fw	CCCGGGATGGTCTCTGCGATCACCAG
3UTR_*Spe*I_rev	ACTAGTGCAGCAGTTTGATAGTTATCCC
*ptrA*_southern_fw	GCACTGAACCCATTCGGGTAGTGAG
*ptrA*_southern_rev	CGGTGTTCGTTCCCAGTCATCG

Supplementary Figures

```
1   ATGCATATTACTAGCATTGCCATTGTCTTCTTCGCCGCAATGGGCGCGGTTGCTAGCCCCATCGCGACCGAGTCG
1   M  H  I  T  S  I  A  I  V  F  F  A  A  M  G  A  V  A  S  P  I  A  T  E  S
1   ATGCATATTACTAGCATTGCCATTGTCTTCTTCGCCGCAATGGGCGCGGTTGCTAGCCCCATCGCGACCGAGTCG

76  GACGATCTTGATGCCCGAGACGTACAGCTTAGTAAATTCGGAGGAgtaagttcttcttataagatgtctatatag
26  D  D  L  D  A  R  D  V  Q* L  S  K  F  G  G
76  GACGATCTTGATGCCCGAGACGTACAGCTTAGTAAATTCGGAGGA------------------------------

151 aaatagcactaacctttctgaaccgctttacagGAATGCAGCTTGAAACACAACACGTGCACATACCTAAAGGGT
41                                   E  C  S  L  K  H  N  T  C  T  Y  L  K  G
121 ---------------------------------GAATGCAGCTTGAAACACAACACGTGCACATACCTAAAGGGT

226 GGAAAGAACCATGTAGTCAATTGCGGTTCGGCCGCCAACAAGAAGgtagattccgattcgattcggggccaattg
55  G  K  N  H  V  V  N  C  G  S  A  A  N  K  K
163 GGAAAGAACCATGTAGTCAATTGCGGTTCGGCCGCCAACAAGAAG------------------------------

301 atttgttcttatcatttaatcttcatctacagTGCAAGTCTGATCGCCACCACTGTGAATACGATGAGCACCACA
70                                  C  K  S  D  R  H  H  C  E  Y  D  E  H  H  K
208 --------------------------------TGCAAGTCTGATCGCCACCACTGTGAATACGATGAGCACCACA


376 AGAGGGTTGACTGCCAGACCCCAGTTTGA
85    R  V  D  C  Q  T  P  V  STOP
251 AGAGGGTTGACTGCCAGACCCCAGTTTGA
```

Figure S1. The nt sequence of the *pafB* gene and the aa sequence of the gene product. Genomic DNA (upper-case), cDNA (italics, upper-case) and deduced aa sequence (bold, upper-case) are shown. The nt numbering starts at the A of the start codon. Non-coding sequences are shown in lower-case letters. Underlined sequences indicate the consensus 5'-splice donor site (gt) and 3'-splice acceptor site (ag) of the introns. The lariat-forming consensus sequence RCTRAC (R = A or G) is underlined by broken lines. The stop codon is marked with a "STOP". The aa residues 1- 34* comprise the prepro-sequence of PAFB.

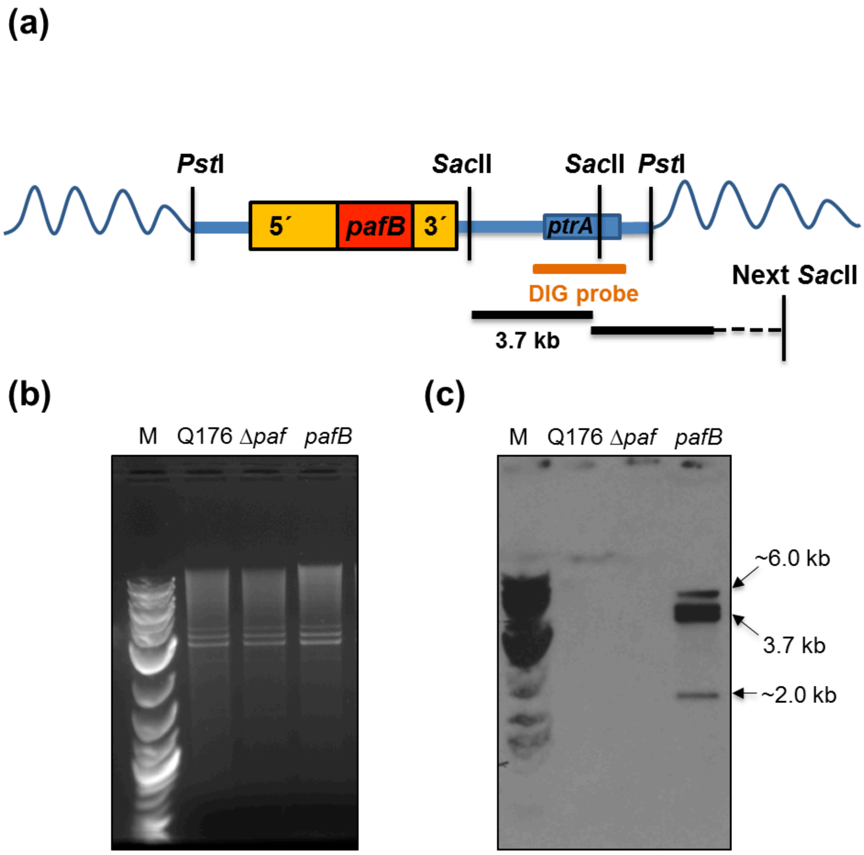

Figure S2. Southern blot analysis for genomic integration of the *pafB* expression cassette in *P. chrysogenum* Δ*paf*. **(a)** Scheme of the expression cassette: *paf*-specific 5'-UTR and 3'-UTR (orange boxes) and *pafB* coding sequence (red box); pSK275 plasmid backbone (blue straight line); *ptrA* resistance cassette (blue box); *P. chrysogenum* genome (blue wavy line); *Pst*I and *Sac*II endonuclease restriction sites; specific DIG-labelled probe (orange line); hybridizing fragment(s) with the expected sizes (black). **(b)** Agarose (0.7% (w/v)) gel electrophoresis for size fractionation of digested genomic DNA of *P. chrysogenum* Q176, *P. chrysogenum* Δ*paf* (both controls) and *P. chrysogenum pafB*. **(c)** Southern blot showing random genomic integrations of the expression cassette. As a size marker, 2-log marker (New England Biolabs) was used.

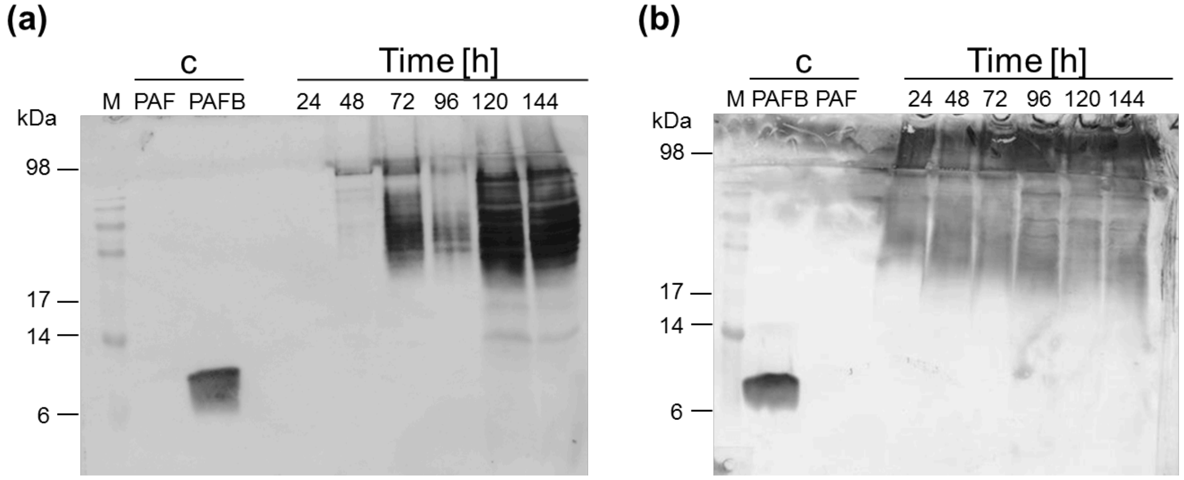

Figure S3. Detection of PAFB in *P. chrysogenum* Q176 cultures. Samples were taken after 24, 48, 72, 96, 120 and 144 h of incubation at 25 °C. Twenty-five µL of 10-fold concentrated **(a)** culture supernatants and 25 µL of **(b)** cell extracts were loaded per lane, size fractionated on an 18% (w/v) SDS-acrylamide gel and transferred on nitrocellulose membranes for Western blot detection. Polyclonal antibodies specific for PAFB and PAF, respectively, were used for protein detection. c, purified PAFB or PAF (1 µg, respectively) were loaded as controls.

(a)

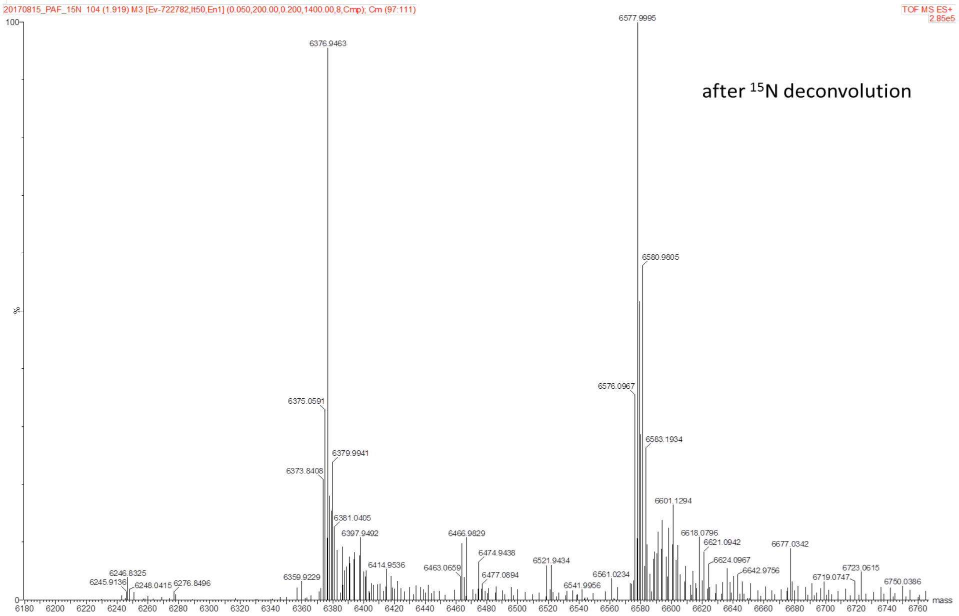

(b)

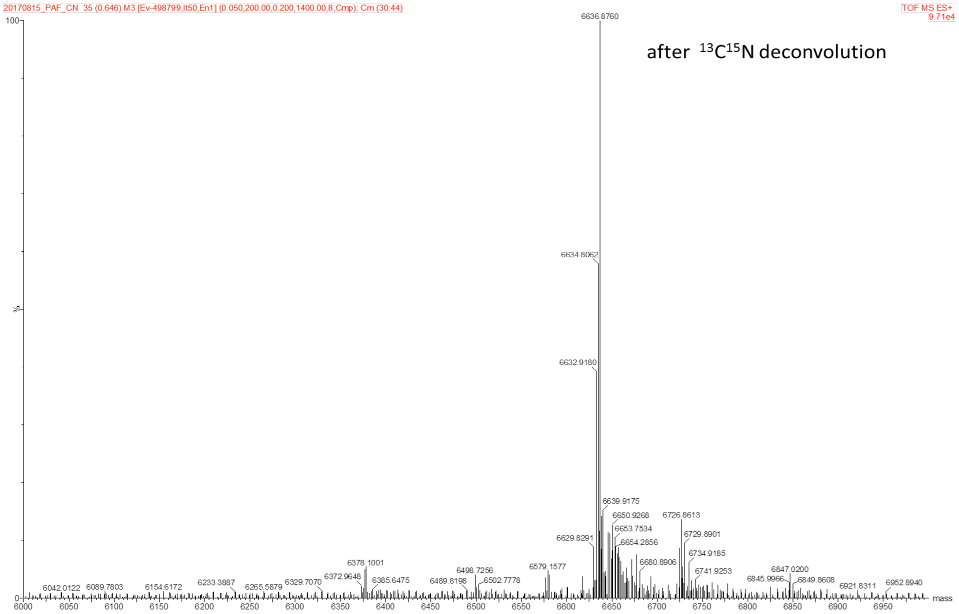

Figure S4. Time-of-flight MS-ES spectra of the isotope labelled PAFB samples as used for NMR studies. **(a)** ^{15}N-labelled sample, **(b)** ^{13}C/^{15}N labelled sample. Original spectra are software deconvoluted in order to provide masses independent of ionic charges and the impact of natural abundance NMR active nuclei. The ^{15}N labelled sample showed two components with the following mono-isotopic masses: PAFB: 6578 (6577.8, if calculated for ^{15}N/^{13}C = 99 % / 0 %) and sfPAFB: 6376.9 (6375.8, if calculated for ^{15}N/^{13}C = 99 % / 0 %), while the ^{15}N/^{13}C labelled sample consists of nearly exclusively the sfPAFB: 6636.9 (6637, if calculated for ^{15}N/^{13}C = 99 % / 99 %).

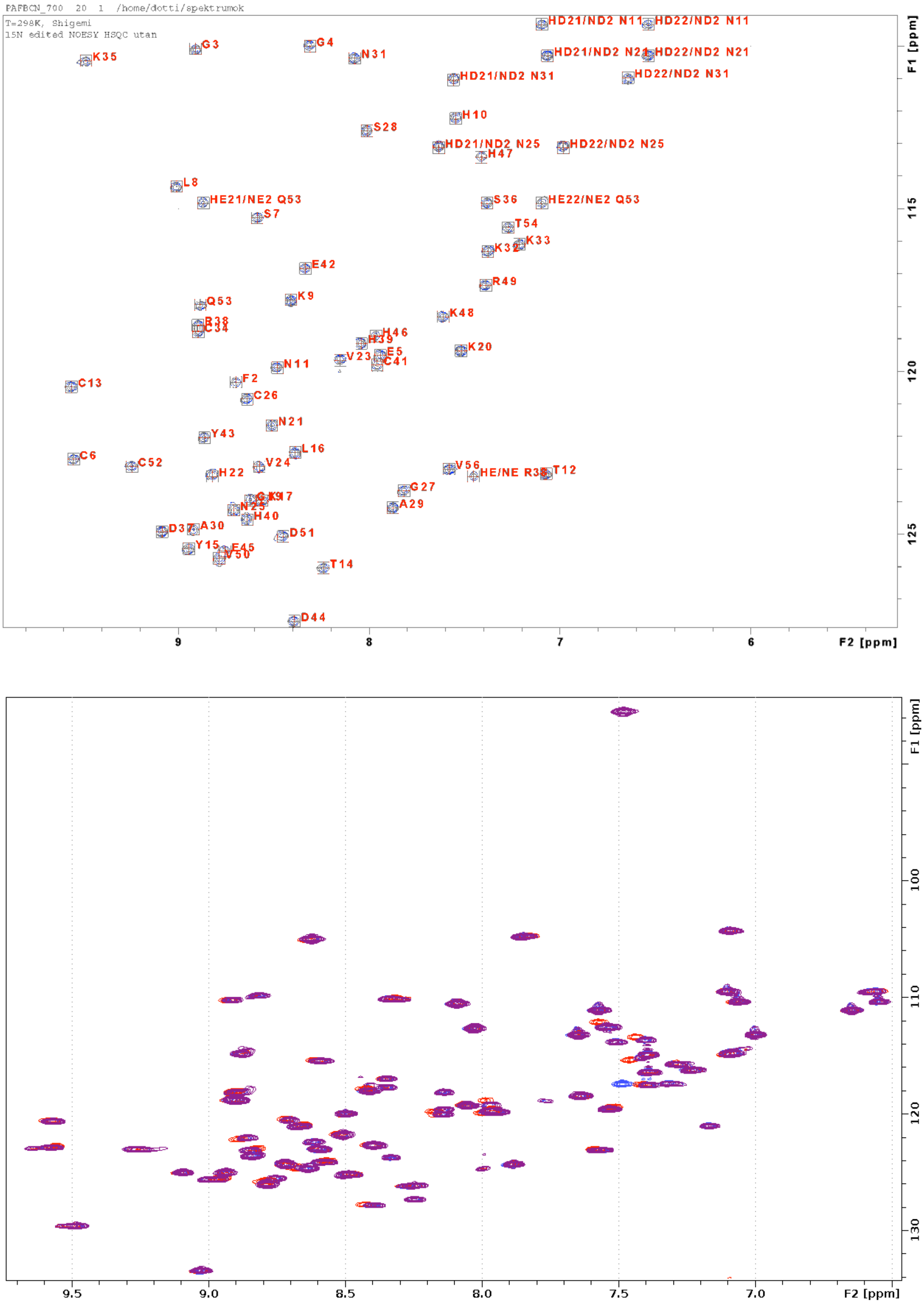

Figure S5. Top: ^{1}H-^{15}N HSQC spectrum of ^{13}C, ^{15}N-labelled PAFB with the assigned peaks labelled. This sample contains the sfPAFB form as dominant species. Bottom: superimposed ^{1}H-^{15}N HSQC spectra of ^{15}N- (blue peaks) and ^{13}C, ^{15}N-labelled (red peaks) PAFB. For the doubly labelled form the resonances for the 3 N-terminal resonances are missing, suggesting that in this case the sfPAFB form is dominant. The excellent overlap between the spectra and the number of peaks reveals that there are no different conformations present in the two structures, indicating that the presence (or altered dynamics) of the N-terminal 2 residues has no significant effect on the overall structure of the protein.

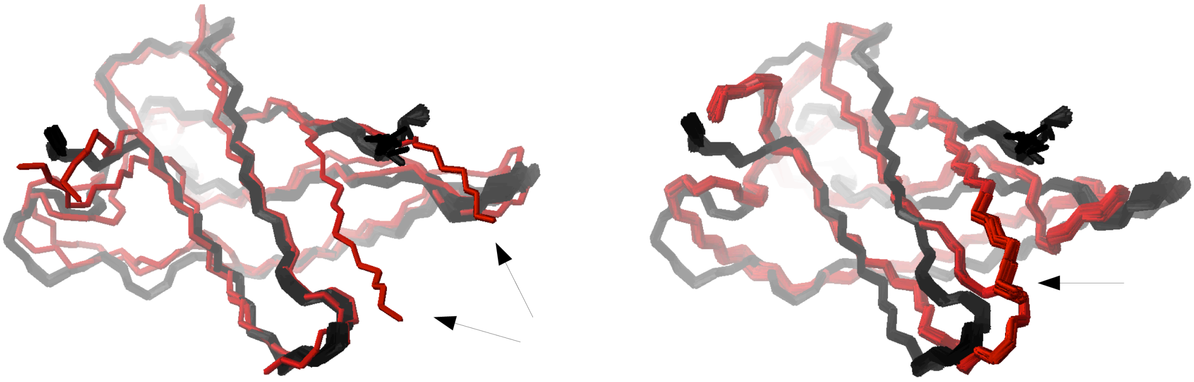

	L *S* K F G G E C S L K H N T C T Y L K G G K N H V V N C G S A A N K K C K S D R H H C E Y D E H H K R V D C Q T P V
sfPAFB	L L L E E E L S S S L E E E E E S S S L E E E E E L L L S S S S S L S L T T L E E E E E T T T T E E E L S L L L
PAFB MD	*L* *L* E E E E E E E T T T T E E E E E S S S S E E E E E L L L L T T T L L B S T T L E E E E E T T T L L E E E E L L L
PAFB NOE-restrained MD	*L* *L* L E E E E E E T T T L E E E E E E T T E E E E E E L L L L T T T L L S L T T E E E E E E T T T T E E E E S L L L

Figure S6. Structure of the N-terminus of the modeled full-length PAF structures. Top left: superimposed structures of sfPAFB (Protein Data Bank ID 2NC2, black) and the NOE-restrained two final conformers of the full-length PAFB model (red). Top right: superimposed structures of sfPAFB and the last 30 conformers from a typical 3 ns MD run (red). The N-termini in the different conformers are marked with arrows. Bottom: secondary structure annotation of the structures as calculated with DSSPcont. The N-terminal extension is capable of adopting an extended β-strand conformation (orange) in both types of simulations.

(a)

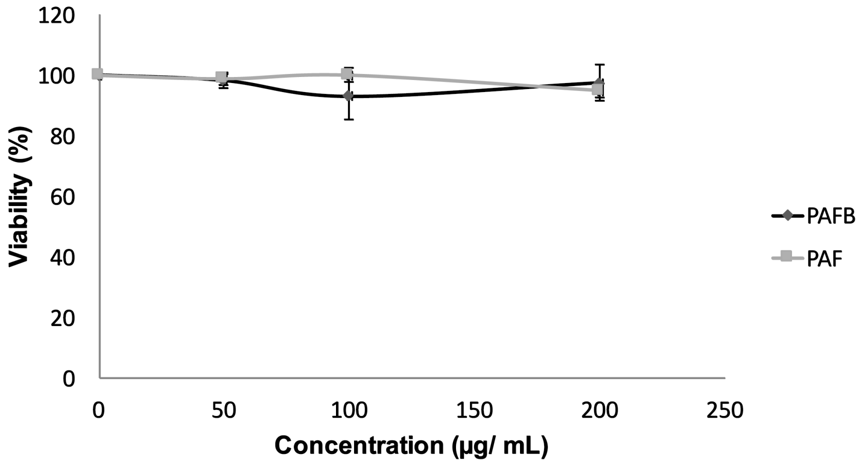

(b)

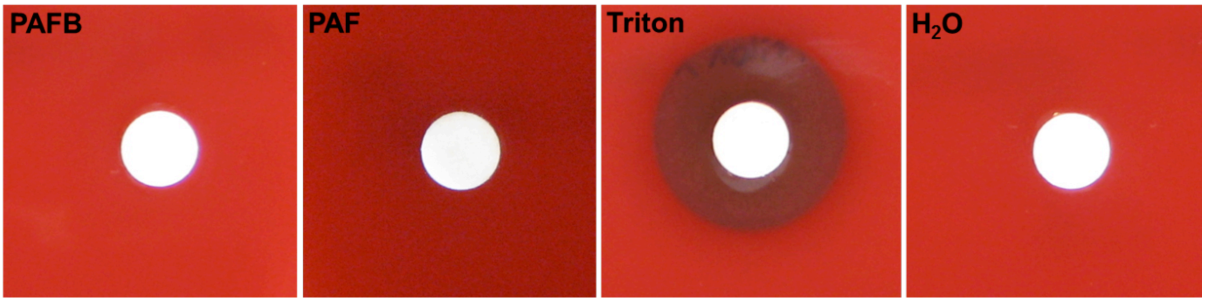

Figure S7. Assessment of PAFB toxicity on mammalian cells *in vitro*. **(a)** Viability given in (%) of human epithelial cells L132 treated with increasing concentrations of PAFB and PAF. Untreated L132 cells served as control (100% viability) **(b)** Haemolytic activity of PAFB on sheep erythrocytes in Columbia Blood Agar plates. Ten µL of PAFB (300 µM), PAF (300 µM), 0.2 × Triton X-100 (positive control) and H_2O (negative control) were pipetted on filter disks and the plates were incubated at 37 °C for 24 h.

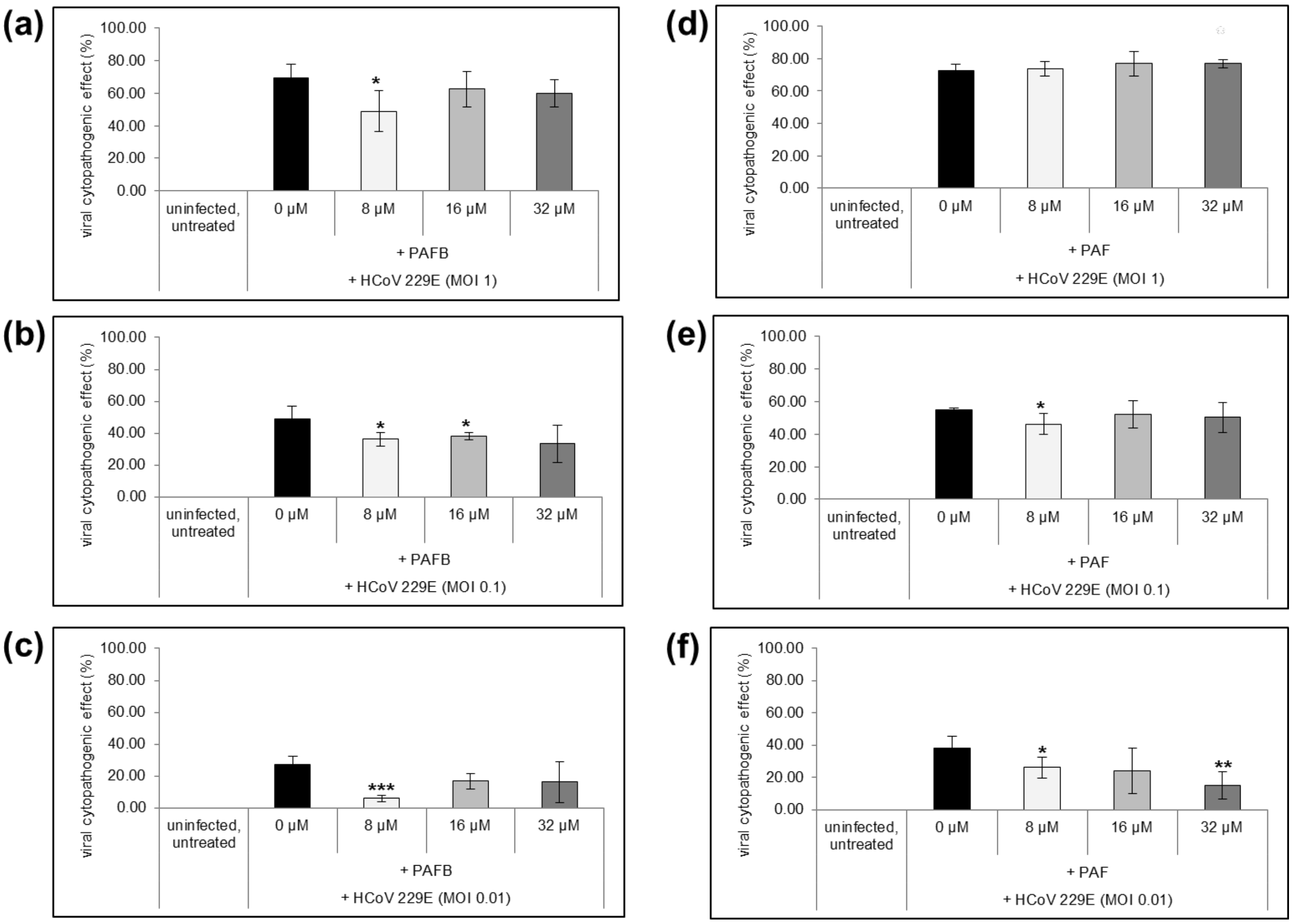

Figure S8. Impact of PAFB and PAF on the CPE of HCoV 229E - infected L132 cells. Proteins were applied in the concentration range 0-32 µM, respectively at **(a,d)** MOI 1, **(b,e)** MOI 0.1 and **(c,f)** MOI 0.01.

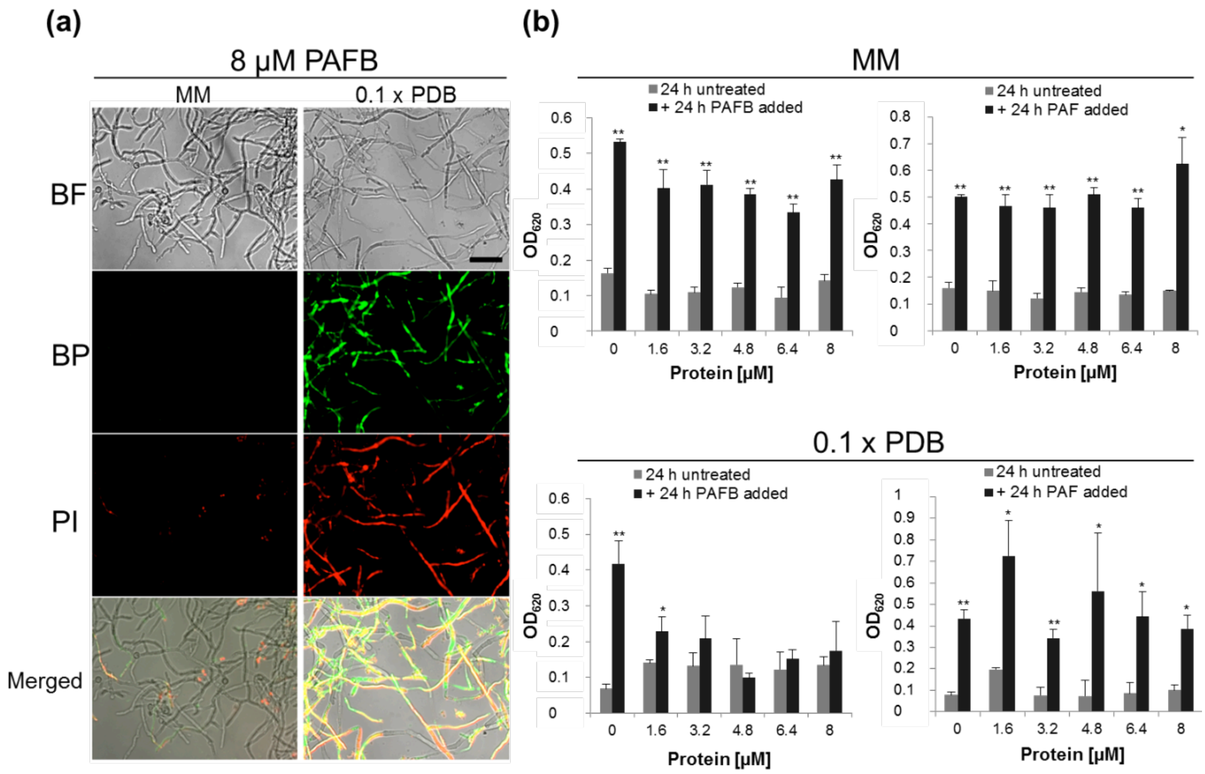

Figure S9. Medium-dependent PAFB activity in *P. chrysogenum* submerse culture. **(a)** Uptake of BODIPY-PAFB (8 µM) in 24 h-old *P. chrysogenum* mycelia grown in MM or 0.1 × PDB. Mycelia were exposed to BODIPY-PAFB for 2.5 h, then co-stained with PI for 10 min before imaging. **(b)** Mycelial growth in the presence of PAFB or PAF in MM and 0.1 × PDB, respectively. *P. chrysogenum* was grown in the respective media for 24 h to reach an OD_{620} of 0.1-0.2 (grey columns). Then PAFB and PAF were added at increasing concentrations and after an additional 24 h incubation, the growth was determined spectroscopically (black columns). The values in the charts represent the mean ± SD OD_{620} (n = 3). *$p < 0.05$, **$p < 0.001$. BF = Brightfield, BP = BODIPY-labelled PAFB. Scale bar = 30 µm.

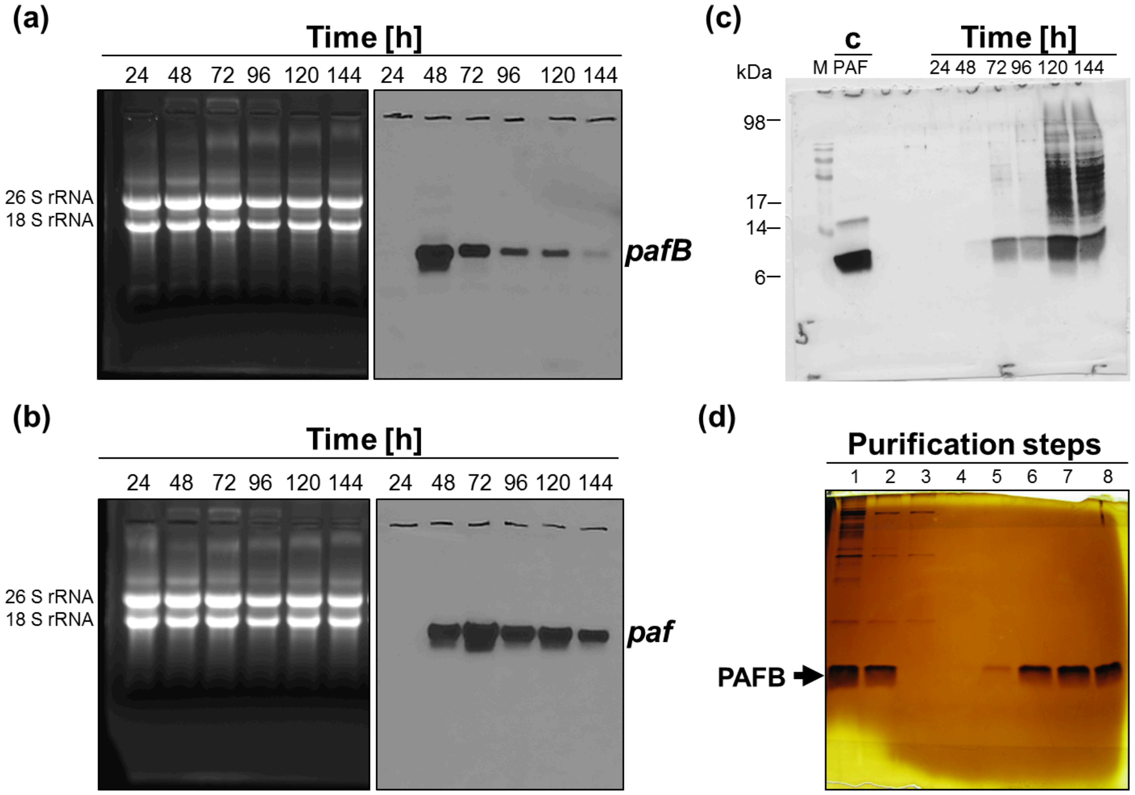

Figure S10. Full length gels and blots of Fig. 1 and Fig. 3. **(a)**, **(b)** For Northern blot analysis 10 µg total RNA of *P. chrysogenum* Q176, cultivated at 25 °C for the indicated time points, were loaded per lane on a 1.2% (w/v) denaturing agarose gel, blotted and hybridized with a *pafB* **(a)** or **(b)** *paf* specific DIG-labelled probe. Ethidium bromide-stained 26S and 18S rRNA is shown as loading controls. **(c)** For Western blot analysis, 25 µL of 10-fold concentrated culture supernatants were loaded per lane, size fractionated on a 18% (w/v) SDS-polyacrylamide gel and transferred on a nitrocellulose membrane. A polyclonal antibody was used for specific PAF detection. c, purified PAF (1 µg) was loaded as a control. **(d)** Silver stained SDS-polyacrylamide gel (18% (w/v)) with samples of PAFB purification steps (25 µL per lane). 1, crude supernatant of 96 h-old culture; 2, ultra-filtrated culture broth (<30 kDa); 3, flow through of cation exchange chromatography; 4, column wash fraction; 5 – 8, elution fractions containing pure PAFB. For clarity reasons, for this study irrelevant lanes are not shown in (a), (b) and (d).

Supplementary References

1 Sonderegger, C. *et al.* A *Penicillium chrysogenum*-based expression system for the production of small, cysteine-rich antifungal proteins for structural and functional analyses. *Microb Cell Fact* **15**, 192, doi:10.1186/s12934-016-0586-4 (2016).

2 Zadra, I., Abt, B., Parson, W. & Haas, H. *xylP* promoter-based expression system and its use for antisense downregulation of the *Penicillium chrysogenum* nitrogen regulator NRE. *Appl Environ Microb* **66**, 4810-4816, doi:Doi 10.1128/Aem.66.11.4810-4816.2000 (2000).

3 Kleywegt, G. J. J. A. A super position. *ESF/CCP4 Newsletter Protein Crystallogr* **31**, 9–14 (1994).

4 Hess, B., Kutzner, C., van der Spoel, D. & Lindahl, E. GROMACS 4: algorithms for highly efficient, load-balanced, and scalable molecular simulation. *J Chem Theory Comput* **4**, 435-447, doi:10.1021/ct700301q (2008).

5 Richter, B., Gsponer, J., Várnai, P., Salvatella, X. & Vendruscolo, M. The MUMO (minimal under-restraining minimal over-restraining) method for the determination of native state ensembles of proteins. *J Biomol NMR* **37**, 117-135, doi:10.1007/s10858-006-9117-7 (2007).

6 Fizil, Á., Gáspári, Z., Barna, T., Marx, F. & Batta, G. "Invisible" conformers of an antifungal disulfide protein revealed by constrained cold and heat unfolding, CEST-NMR experiments, and molecular dynamics calculations. *Chemistry* **21**, 5136-5144, doi:10.1002/chem.201404879 (2015).

7 Lindorff-Larsen, K. *et al.* Improved side-chain torsion potentials for the Amber ff99SB protein force field. *Proteins* **78**, 1950-1958, doi:10.1002/prot.22711 (2010).

8 Onufriev, A., Case, D. A. & Bashford, D. Effective born radii in the generalized born approximation: the importance of being perfect. *J Comput Chem* **23**, 1297-1304, doi:10.1002/jcc.10126 (2002).

9 Andersen, C. A., Palmer, A. G., Brunak, S. & Rost, B. Continuum secondary structure captures protein flexibility. *Structure* **10**, 175-184 (2002).

10 Lupyan, D., Leo-Macias, A. & Ortiz, A. R. A new progressive-iterative algorithm for multiple structure alignment. *Bioinformatics* **21**, 3255-3263, doi:10.1093/bioinformatics/bti527 (2005).

11 Koradi, R., Billeter, M. & Wüthrich, K. MOLMOL: a program for display and analysis of macromolecular structures. *J Mol Graph* **14**, 51-55, doi: 10.1016/0263-7855(96)00009-4 (1996).

12 Baker, N. A., Sept, D., Joseph, S., Holst, M. J. & McCammon, J. A. Electrostatics of nanosystems: application to microtubules and the ribosome. *Proc Natl Acad Sci USA* **98**, 10037-10041, doi:DOI 10.1073/pnas.181342398 (2001).

13 Schrödinger Release 2017-2: Maestro, S., LLC, New York, NY, 2017.

14 Mosmann, T. Rapid colorimetric assay for cellular growth and survival: application to proliferation and cytotoxicity assays. *J Immunol Methods* **65**, 55-63 (1983).

15 Rodríguez-Martín, A. *et al.* Characterization of the novel antifungal protein PgAFP and the encoding gene of *Penicillium chrysogenum*. *Peptides* **31**, 541-547, doi:10.1016/j.peptides.2009.11.002 (2010).

16 Gasteiger, E. *et al.* Protein identification and analysis tools on the ExPASy server in *The proteomics protocols handbook* (ed. Walker, J. M.) 571-607 (Humana Press 2005).

17 Maurer, E. *et al.* *Galleria mellonella* as a host model to study *Aspergillus terreus* virulence and amphotericin B resistance. *Virulence* **6**, 591-598, doi:10.1080/21505594.2015.1045183 (2015).

18 Hegedűs, N., Sigl, C., Zadra, I., Pócsi, I. & Marx, F. The *paf* gene product modulates asexual development in *Penicillium chrysogenum*. *J Basic Microbiol* **51**, 253-262, doi:10.1002/jobm.201000321 (2011).
